# Defining nature, responses and drivers of regulatory- and conventional CD4^+^ T cells in the human term decidua

**DOI:** 10.3389/fimmu.2026.1865340

**Published:** 2026-08-04

**Authors:** Lotte J. Verleng, Julia Busselaar, Mark Mensink, Ellen Schrama, Hanneke Kapsenberg, Carin van der Keur, Xin Lei, Michael Eikmans, Yanling Xiao, Sander de Kivit, Jannie Borst

**Affiliations:** 1 Department of Immunology, Leiden University Medical Center, Leiden, Netherlands; 2 Oncode Institute, Leiden University Medical Center, Leiden, Netherlands

**Keywords:** decidua, effector differentiation, maternal-fetal tolerance, non-lymphoid tissue, T-cell response, transcriptomics, Treg

## Abstract

**Background:**

During mammalian pregnancy, a maternal immunoregulatory network develops in the decidua that fosters fetal development, maintains tolerance to fetal antigens and protects against infection. Herein, decidual regulatory T cells (Tregs) are crucial for pregnancy success.

**Aim and methodology:**

To understand the nature and response of maternal CD4^+^ Tregs and CD4^+^ conventional T-cells (Tconv) in healthy human pregnancy, we analyzed these cells in decidua and blood at term, caesarean delivery by single-cell transcriptomics and TCR sequencing.

**Results:**

Data mining revealed novel discerning, as well as shared features and functionalities of decidual CD4^+^ Tregs and Tconvs. Both cell types showed evidence of antigen recognition and activation in the decidua, followed by local clonal expansion and effector differentiation. Tregs were largely of the thymus-derived (t)Treg lineage that has a self-antigen reactive T cell receptor (TCR) repertoire. Tregs locally expanded, more so than Tconvs, and differentiated into typical non-lymphoid tissue (NLT)-resident effector cells with discerning cell surface markers and multiple suppressive functions, driven by TNF receptor-2 costimulation. Additional factors, including IFNs, interleukins and prolactin emerged as shared drivers of decidual Treg and Tconv effector differentiation. Effector Tconvs had cell lineage-discerning proinflammatory and cytotoxic capacities, restrained by cell-intrinsic and -extrinsic mechanisms, while sharing glycolytic and antigen-presenting features with Tregs. Treg and Tconv subpopulations showed signs of exhaustion, suggesting chronic antigenic stimulation. Overall, the observed features argue for ongoing *de novo* T-cell priming and dynamic T-cell turnover in the decidua throughout pregnancy.

**Conclusion:**

Our findings suggest that non-self-reactive CD4^+^ Tconvs and self-reactive tTregs are continuously primed in lymphoid organs during pregnancy, reactivated by antigen in the decidua and dynamically turned over. In the decidua, CD4^+^ Tconvs and Tregs expand and differentiate under influence of specific cytokines into tissue-resident effector cells with opposing and restrained pro- and anti-inflammatory functions, as well as shared antigen-presenting capacity.

**Significance:**

These molecular definitions of decidual CD4^+^ Tregs and Tconvs can be used to aid diagnostics at mRNA and protein level. Moreover, they facilitate mechanistic understanding of maternal-fetal tolerance, based on extrapolation of known T-cell lineage characteristics and tissue adaptations in health and disease.

## 1 Introduction

During mammalian pregnancy, the blastocyst implants into the uterine endometrium and develops into both fetus and placenta. The uterine endometrium transforms into decidual tissue, which supports fetal and placental development. Fetal cytotrophoblasts (CTBs) form placental villi covered by further differentiated syncytiotrophoblasts (STBs), while the inner CTB layer anchors the placenta to the decidua through invasion (1). CTBs also differentiate into extravillous trophoblasts (EVTs), which remodel maternal blood vessels to enable direct maternal-fetal contact and efficient exchange of nutrients, gases and waste products (2). The decidua basalis (DB) constitutes the placental interface, while the decidua parietalis (DP) lines the distal uterine wall. This type of placentation occurs in mammals with a hemochorial placenta, including humans and mice (3), and establishes the structural context for maternal-fetal immune interactions (4, 5).

The immune cell composition of the endometrium dynamically changes upon decidualization during the menstrual cycle and pregnancy, in response to seminal fluid and fetal signals. The decidua is populated with maternal macrophages, dendritic cells (DCs) and lymphoid cells, but direct recognition of the semi-allogeneic fetal trophoblasts by T cells is limited because STBs do not express any major histocompatibility complex (MHC) molecules and EVTs only express HLA-C. Furthermore, EVTs attenuate natural killer (NK) cell activity via non-polymorphic HLA-E and -G (6, 7) and T-cell activation via PD-1 ligands, which is critical for successful pregnancy (8). Indirect recognition of the allogenic fetus by T cells is possible, however, also on basis of MHC-II, because macrophages and DCs of the mother will be able to phagocytose fetal cell debris and present antigens. Responses of CD4^+^ and CD8^+^ Tconvs may be required in case of fetal infection and therefore Tconv responses must be carefully controlled. Accordingly, Tregs are required for fetal tolerance from the first trimester onwards, as shown by Treg depletion studies in mice (9–13) and human data linking low Treg number or impaired Treg function in decidua and peripheral blood to pregnancy failure (14, 15).

Tregs are required for allogeneic but not syngeneic pregnancy success (9), indicating that decidual Tregs restrain Tconv responses to paternal alloantigens. Indeed, mouse studies showed that pregnancy induces maternal T-cell tolerance to paternal (MHC) molecules, allowing acceptance of the fetus as well as implanted tumors of the same MHC type (16). Paternal antigen presentation to maternal CD4^+^ and CD8^+^ Tconvs relies on maternal antigen presenting cells (17), which may include conventional (c)DCs (18). Trafficking of cDCs to draining lymph nodes (dLNs) typically initiates T-cell priming (19), but upon decidualization, DCs appear physically trapped (20), and lymphatic vessels disappear (21), suggesting an additional mechanism preventing anti-fetal T-cell priming. Nevertheless, Tconv responses to paternal antigens have been demonstrated in mouse models and in humans (17, 22–25). During murine pregnancy, Tregs were shown to expand in uterine dLNs and systemically, with more overt responses in the allogeneic setting (12). In human pregnancy, Treg frequencies among decidual T cells also increase (12, 26), particularly upon HLA-C mismatch (27). Although these data suggest that the responding Tregs are activated by paternal allo-antigens, this has not been proven (28). Alternatively, Tregs may be maternal self-antigen specific and expand in response to IL-2 produced by activated (alloreactive) Tconvs (29).

A key question has been whether decidual Tregs are thymus-derived (t)Tregs, peripherally-induced (p)Tregs or both. While tTregs are generated as a separate cell lineage in the thymus, pTregs arise during an ongoing immune response from CD4^+^ Tconvs (30, 31). Genetic evidence from a mouse study has argued in favor of pTregs being at least in part responsible for pregnancy success (32). In the studies cited above, Tregs were mostly defined on a CD4^+^CD25^high^ phenotype rather than expression of Forkhead Box P3 (FOXP3), which has been used later to define decidual Tregs (33–35). The core transcriptomic signature of tTregs includes *FOXP3*, *IKZF2/4*, *CTLA4*, *IL2RA/RB*, *TNFRSF4, -9* and *-18* (36–40). FOXP3 installs part of this signature (36), while chromatin remodeling and transcription factors such as IKAROS Family Zinc Finger 2 (IKZF2; HELIOS) and IKZF4 (EOS) further stabilize tTreg identity (29, 41). It is important to classify Tregs as tTregs or pTregs because tTregs have a primarily self-reactive TCR repertoire and stably express FOXP3 (42), while pTregs recognize non-self antigens and may lose FOXP3 expression. Loss of the *Foxp3* gene in established Tregs results in loss of their *in vivo* suppressive function and a gained ability to produce IL-2 and pro-inflammatory cytokines (43). Because tTregs cannot produce IL-2, they depend on Tconv-derived IL-2 for clonal expansion and survival (42). Importantly, they retain their suppressive identity in pro-inflammatory environments by differentially processing cytokine signals compared to Tconvs (44).

Tregs populate non-lymphoid tissues (NLT) both in health and disease, where they display a unique, tissue-resident phenotype and gene expression profile (45–47). The relationship of NLT-resident effector (e)Tregs to naive tTregs has long been unclear, but we recently demonstrated that naive tTregs from human blood can differentiate into cells with the NLT-resident gene expression profile upon TCR-mediated activation and costimulation via TNF receptor (TNFR)2 (48). Accumulating evidence indicates that, both in the first trimester and at term, the human decidua is primarily populated by Tregs with unique TCR repertoires compared to decidual Tconvs, suggesting their divergent origins (34, 35). In addition, decidual Tregs display an effector phenotype resembling Tregs in other peripheral tissues, including tumors (33, 35). Importantly, a CCR8^+^ decidual Treg population has been implicated in promoting pregnancy success in mice (35), highlighting a critical role for specialized Treg subsets at the maternal-fetal interface (47).

With these insights in mind, we defined discerning and common features of human decidual CD4^+^ Tregs and Tconvs, as isolated from the placenta at term delivery by scRNA- and TCRseq analysis. CD4^+^ Tregs and Tconvs from matched maternal peripheral blood and cord blood were also included in the analysis. The donors underwent healthy pregnancies with delivery by caesarean (C-)section, excluding immune regulatory effects of labor. We resolved the clonal relationships among and between Tregs and Tconvs, their activation states and functional properties, revealing a dynamic immune landscape in which maternal CD4^+^ T cells undergo continuous priming and reactivation in the decidua, followed by local expansion and effector differentiation. Our findings indicate that decidual Tregs are primarily tTregs, based on non-overlapping TCR repertoires compared to Tconvs and enrichment of the stable tTreg core signature (44). These tTregs adopt an NLT-resident gene expression profile within the decidua, under influence of TNFR2 driver signals. Effector Tconvs and Tregs shared capacity for antigen presentation on MHC-I and II, while CD4^+^ Tconvs become vigilant cytotoxic and pro-inflammatory cells, apparently restrained intrinsically by inhibitory receptors and extrinsically by Tregs. Our findings suggest a dynamic local immune regulatory network in the decidua, that maintains maternal-fetal tolerance whilst being alert to combat infection. This study classifies the great majority of decidual Tregs at term pregnancy as tTregs, provides important phenotypic distinctions between decidual CD4^+^ Tregs and Tconvs and clarifies their response to the fetus in terms of clonal expansion, acquisition of distinct functional states and turnover.

## 2 Materials and methods

### 2.1 Isolation of lymphocytes from human blood and placenta

Donors were recruited at Leiden University Medical Center and clinical parameters are listed in **Table 1**. Maternal (m)PB was collected 1 h prior to C-section and umbilical cord blood (UCB) was collected directly after, in heparin anticoagulation blood tubes. Mononuclear cells were isolated from mPB and UCB by Ficoll-Paque (GE Healthcare) density gradient centrifugation (820x *g*, no brake, room temperature, 20 min). Mononuclear cells were isolated from DP and DB as described (49), with adaptations as follows: DP and DB were macroscopically dissected and tissues were washed in PBS, minced, and resuspended in RPMI 1640 (Life Technologies) with 0.2 mg/ml Collagenase IV (MilliporeSigma) and 0.01 mg/ml DNAse I (Sigma) at a ratio of 10 ml digestion mix per 5 g tissue. Tissue was homogenized with a GentleMACS tissue dissociator (Miltenyi Biotec), using program m-tumor 02 (37 seconds at 235 rpr) for DP and program m-heart 02.01 (17 seconds at 668 rpr) for DB and incubated for 30 min at 37°C. Next, cell suspensions were sequentially passed through a 250-μm- and a 70-μm filter and washed in RPMI 1640 with 5% FCS. They were mixed with 20 ml of 1.023 g/ml Percoll (GE Healthcare) and separated by density gradient centrifugation (820*g*, no brake, room temperature, 30 min) on a Percoll gradient (10 ml of 1.080 g/ml, 15 ml of 1.053 g/ml, pH = 7.4). Mononuclear cells were isolated from the 1.080–1.053 g/ml interface. Subsequently, samples were washed twice in RPMI. Cells were processed for flow cytometric analysis directly after isolation, or cryopreserved and stored in liquid nitrogen until processing for scRNAseq. Decidual and mPB cells were by definition from females, while UCB was from males or females as indicated in **Table 1**.

**Table 1:** Characteristics of patients included in the study.

Donor #^1^	Maternal age	BMI^2^	Gravidity^3^	Parity^4^	Gestational age	Gender baby^5^	C-section indication	Analysis
1	32 y	25.6	3	1	39 w	F	Previous C-section	scRNA/TCRseq^6^
2	31 y	29.0	2	1	39 w + 1 d	M	Previous C-section	scRNA/TCRseq^6^
3	31 y	22.0	2	1	40 w + 5 d	F	Previous C-section	scRNA/TCRseq^6^
4	33 y	24.7	1	0	39 w + 1 d	M	Caput in fundo	FC^7^
5	30 y	30.6	1	0	39 w + 4 d	M	Caput in fundo	FC^7^
6	41 y	30.1	1	0	37 w + 6 d	F	Placenta praevia	FC^7^
7	29 y	27.0	4	1	39 w + 2 d	F	Previous C-section	FC^8^
8	33 y	32.4	3	2	38 w	F	Previous C-section	FC^8^
9	34 y	25.4	2	1	38 w + 2 d	F	No C-section	FC^8^
10	40 y	18.6	5	2	35 w + 5 d	F	Previous C-section; PE	FC^8^

^1^ Donor 1–5, 7 and 8: spontaneous conception, Donor 6 and 10: intracytoplasmic sperm injection, Donor 9: IVF. Donor 8 had diabetes gravidarum; ^2^BMI, body mass index (prior to pregnancy); ^3^Total number of pregnancies, prior to this study; ^4^Total number of pregnancies that reached viable gestational age, prior to this study. Donor 1 had one miscarriage and donors 7 and 10 had two miscarriages in the past; ^5^F, female, M, male; ^6^see **Figures 3**–**7**, and **Supplementary Figures 1**–**3** and **5**–**7**; ^7^see **Figure 1** and **Supplementary Figure 1** FC, flow cytometry; ^8^see **Supplementary Figure 4** and Mensink et al., 2024 (48). (PE, preeclampsia).

### 2.2 Phenotypic analysis by flow cytometry

**Figure 1:**
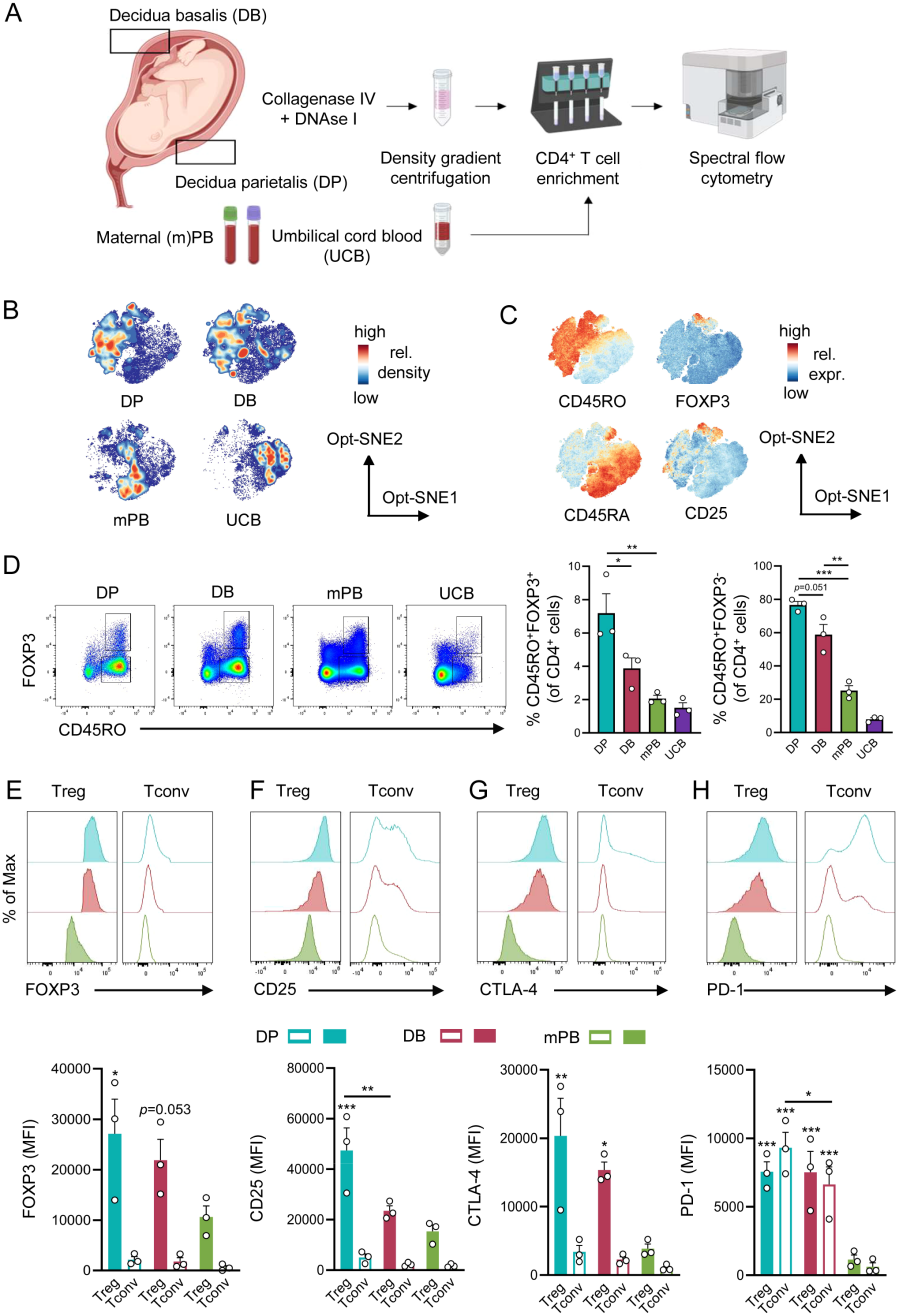
Flow cytometric analysis of CD4+ T cells from decidua and blood. **(A)** Methodology used for isolation CD4^+^ T cells from DB, DP, mPB and UCB prior to analysis by spectral flow cytometry. Created in BioRender (https://BioRender.com/6gve59e). **(B, C)** Opt-SNE cluster analysis of concatenated flow cytometric data from all CD4^+^ T-cell samples, indicating relative signal density in the different tissues **(B)** and relative expression of indicated markers **(C)**. **(D)** Representative dot plots (left) and quantification (right) of CD45RO^+^ effector Treg and Tconv frequencies in the four tissues. One-way ANOVA with Tukey’s *post hoc* test was used for statistical analysis. **(E–H)** Representative histograms (upper panels) and quantification (lower panels) of mean fluorescence intensity (MFI) of FOXP3 **(E)**, CD25 **(F)**, CTLA-4 **(G)** and PD-1 **(H)** measurements on CD4^+^ T cells from the indicated tissues. Two-way repeated measures ANOVA with Tukey’s *post hoc* test was used for statistical analysis (*p < 0.05, **p < 0.01, ***p < 0.001). **(A–H)** Data represent samples obtained from *n* = 3 individual donors (**Table 1**). **(D–H)** Data are presented as mean ± SEM.

Donors used for the scRNAseq (n=3, pooled) and the flow cytometry analyses shown in **Figure 1** and **Supplementary Figure 1** (n=3) had healthy term pregnancies and underwent C-section prior to labor commencement (**Table 1**), while donors (n=4) used for the flow cytometry analysis shown in **Supplementary Figure 4** had a more variable clinical history (**Table 1**). CD4^+^ T cells were isolated from decidua, mPB and UCB by magnetic purification from mononuclear cells by MACS cell separation (Miltenyi Biotec) using CD4 MicroBeads (Miltenyi Biotec, cat. #130-045-101). The CD4^+^ cell fraction was washed in PBS/1% FCS and incubated on ice with human Fc-Block (BioLegend) for 5 min and next stained for 30 min with the mAbs listed in **Supplementary Table 1**. Cell viability was assessed using LIVE/DEAD blue dye (ThermoFisher Scientific). For intracellular staining, cells were fixed and permeabilized using the eBioscience™ FOXP3 transcription factor staining buffer set (Invitrogen), according to the manufacturer’s instructions. Cells were stained for 45 min on ice in permeabilization buffer with the following mAbs: FOXP3-APC (Invitrogen), Helios-PE-Cy7, EOS-PE (both Biolegend) and CTLA-4-BB700 (BD Biosciences). After washing, flow cytometry analysis was performed on a 5-laser Cytek Aurora with SpectroFlo software (Cytek Biosciences). Flow cytometry data were analyzed with the OMIQ platform (Dotmatics) or FlowJo software. In OMIQ analyses, the Flow AI algorithm (50) was used for anomaly detection and exclusion, followed by data scaling and compensation when necessary. Cells of interest were gated as visualized in **Supplementary Figure 1**. Dimension reduction was performed using Opt-SNE (51) using all markers except Live-dead, CD3, CD4 and CD8. Mean expression data were exported for quantification.

### 2.3 Sample and library preparation for scRNAseq and coupled scTCRseq

Mononuclear cells were isolated by density centrifugation from DP, DB, mPB and UCB (n=3, see **Table 1**) and cryopreserved as described above. A small fraction was analyzed by flow cytometry to check frequencies of decidual Tregs which were comparable between donors. Before flow cytometric sorting, cells were quickly thawed in a 37 °C water bath and washed in IMDM with 8% FCS (ThermoFisher Scientific) with 0.01 mg/ml DNAse I (Sigma). Samples were pooled in equal representation of each of the three donors per tissue and stained with CD4-BB700, CD25-PE (BD BioSciences), CD8-PE-Cy7, CD127-BV421 and CD26-APC (BioLegend) mAbs for flow cytometric sorting (**Supplementary Figure 1D**). Cell viability was assessed using LIVE/DEAD blue dye (Invitrogen) for placenta samples and 4-6-diamidino-2-phenylindole (DAPI) stain (Sigma) for blood samples. Tregs (CD25^hi^CD26^low^ in decidua and CD25^hi^CD127^low^ in blood) and Tconvs cells (CD25^low/int^ in decidua and CD25^low^CD127^+^ in blood) were sorted using a BD FACSAria II sorter with FACSDiva Software (version 9.0.1, BD Biosciences). Samples were kept at 4 °C throughout the procedure and collected on ice. After cell sorting, Tregs and Tconvs from each tissue were transferred to a 96-well U-bottom plate (Corning) in a 1:1 ratio to ensure equal representation of both cell types in the scRNAseq data. To discern cells from the different tissues, they were each labeled with a distinct 5’ hashtag oligonucleotide (HTO)-conjugated antibody (TotalSeq™-C0251 - C0254, BioLegend). Cell viability was confirmed to exceed 80% in all samples, whereafter cells from the four tissues were pooled in a 1:1:1:1 ratio in the final sample for single cell isolation and library preparation on the Chromium X platform (10x Genomics). The scRNAseq libraries were prepared using Chromium Next GEM Single Cell 5′ Kit v2, according to the manufacturer’s instructions. Sequencing was performed on a NovaSeq 6000 system (Illumina). TCRαβ sequencing libraries were prepared using the Chromium Single-Cell V(D)J Enrichment Kit for human T cells (PN-100005, 10X Genomics).

### 2.4 Processing of scRNAseq data

Analysis of scRNAseq and scTCRseq was performed using CellRanger (10X Genomics v. 7.1.0) and the Seurat package (version 5.1.0) in R (version 4.3.3). scRNAseq reads were aligned with human reference genome GRCh38.2020.A and barcode counting was performed using ‘cellranger count’. This resulted in a transcriptome of 20,673 cells, with a median sequencing depth of 10,049 reads per cell. HTO demultiplexing was performed using ‘HTODemux’ with a positive quantile of 0.99 (**Supplementary Figures 2A, B**). As a result, each cell was assigned a global HTO classification (‘singlet’, ‘doublet’ or ‘negative’) and a HTO classification (‘Basalis’, ‘Parietalis’, ‘maternal-PBMC’ or ‘UCB’). 12,367 singlets were selected for further analysis using the ‘Subset’ function. Thereafter, variable features were determined using ‘FindVariableFeatures’ with the selection method ‘mean.var.plot’. Data was scaled using ‘ScaleData’ on variable features. The percentage of mitochondrial gene expression per cell was determined using ‘PercentageFeatureSet’ with the pattern “^MT”. Quality control was continued by filtering out cells with <200 or >2500 RNA features, or cells that mapped >10% of their transcripts to mitochondrial genes. Cell cycle scores for G2/M and S phase were assigned, based on expression of G2/M and S phase genes. Data was log-normalized and scaled, whereby G2/M and S scores as well as mitochondrial gene expression were regressed out. Principal component analysis (PCA) was performed on the top 2000 identified variable features using ‘FindNeighbors’ and ‘FindClusters’ (**Supplementary Figure 2C**). Expression of known marker genes was visualized per cluster. Contaminating CD8^+^ T cells, NK cells, B cells and myeloid cells were identified based on *CD3E*, *CD4* and B-cell and myeloid cell lineage markers (**Supplementary Figure 2E**) and discarded from the dataset. TCR sequences were determined using ‘cellranger vdj’ and the reference genome vdj_GRCh38_alts_ensembl_5.0.0. 94.20% of reads was mapped successfully to any V(D)J gene with an estimated 16,535 reads per cell. Together, this resulted in a productive V-J spanning TCRαβ pair for 55% of all singlets, of which 5,514 cells were CD4^+^ (**Supplementary Figure 2F**). TCR and Ig genes were removed from the gene expression count matrix using the ‘grep’ function with the patterns “^TR[ABGD][CVDJ]” and “IG[HKL][CVDJ]” respectively. Subsequently, a Seurat object was created using ‘CreateSeuratObject’ with a minimum of 3 cells expressing each gene and a minimum of 200 features per cell. HTO sequencing data was added as a separate assay to the Seurat object using ‘CreateAssayObject’ and HTO counts were normalized by CLR method using ‘NormalizeData’. Cells without a retrieved TCR sequence were discarded using the ‘Subset’ function, resulting in an object with 5,514 cells. Of these, 4,514 were of ‘tissue’ (DP or DB) origin, and 1,000 were of ‘blood’ (mPB or UCB) origin.

### 2.5 scRNAseq data analysis

*Gene expression analysis* – Data obtained from scRNAseq were processed as described (see **Supplementary Information**). The resulting Seurat object was split into a ‘decidua’ object and a ‘blood’ object. CD4^+^ T-cell clusters were annotated as either ‘Tconv’ or ‘Treg’ based on *FOXP3* expression and additional validation by expression of Treg markers. Marker genes per cluster were identified and genes that were expressed in more than 25% of the cells in a cluster with a log_2_FC threshold >0.5 and an adjusted *p* < 0.05 between two clusters were considered as differentially expressed. Differentially expressed genes (DEG) from all clusters (**Supplementary Table 2**) were visualized in a hierarchically clustered heatmap using the package ‘pheatmap’, whereby relative expression was depicted as z-score for each cluster versus all other clusters (**Supplementary Table 3**). DEGs with similar expression in different clusters were manually annotated and denoted as gene modules and selected genes were visualized in a dot plot using the function ‘DotPlot’ in Seurat. Volcano plots of DEGs between clusters were made in GraphPad Prism.

*Gene ontology (GO) and pathway analysis (IPA) –* DEGs within modules were analyzed for associated biological processes using STRING analysis (https://string-db.org/; v12.0) (52). Functional enrichment visualization was done using the ‘GO: Biological process’ ‘Reactome Pathways’, ‘GO Cellular component’ or ‘KEGG Pathways’ functional categories. Differentially expressed genes identified from scRNA-seq analysis were visualized as an interaction network using STRING, restricting interactions to high-confidence associations (minimum required interaction score >0.7). Network modules were subsequently identified using the built-in *k*-means clustering algorithm. IPA software (Qiagen) (53) was used for functional classification of DEGs as well as upstream regulator analysis. For IPA, all 1152 DEGs were used as input genes, and relative expression values (*z*-scores with a cut-off value < -0.5 or > 0.5) were plotted for 1 cluster versus all. Closely related GO terms were grouped together into functional categories of interest (**Supplementary Table 4**). Upstream Regulator Analysis in IPA was performed on DEGS and z-scores (cut-off value < -0.1 or > 0.1) of cluster 2 versus 5 and cluster 3 versus 7. Upstream regulators were filtered on categories cytokines and growth factors, with a cut-off value of p < 1e-10, and subsequently ordered by Activation z-score. Modules scores of downstream targets were calculated in R using the ‘AddModuleScore’ function, and projected onto the Seurat object.

*Comparative transcriptome analysis –* The genes of the tTreg core signature as identified by Cuadrado et al. (44), the tTreg TNFR2 signature from Mensink et al. (48) (based on the genes with a log_2_FC>0.5), and the top-100 signature genes of CXCL13^+^TCF7^-^ effector/exhausted neoantigen-specific CD4^+^ TILs as identified by Veatch et al. (54) (**Supplementary Table 5**) were used to calculate respective module scores for each cell in our data for comparison. In addition, the genes that defined Treg module I in our dataset (**Supplementary Table 3**) were used to calculate a module score for each cell in the pan-cancer cell atlas by Zheng et al. (55). Violin plots were made for visualization. A one-sample *t*-test with a one-sided *p* < 0.05 was used to test significant enrichment of the module within each cell cluster, using the average module score of all cells in the data set as reference value.

### 2.6 scTCRseq data analysis

Annotated CDR3 and VDJ sequences of paired *TCRA* and *TCRB* transcripts were analyzed using Loupe VDJ Browser (version 5.1.0, 10X Genomics) and projected on UMAP and cluster annotation from Seurat using Loupe browser. For clonotype analysis, only cells that grouped together within their assigned cluster in the UMAP were considered for quantification. Expanded clones were identified as TCR clonotypes containing two or more cells. A χ^2^-test was used to test for association between the number of expanded and non-expanded clones found in Tregs and Tconvs.

### 2.7 Statistical analysis

Statistical analyses, except for scRNAseq analyses, were performed using GraphPad Prism (version 10.2.3). Statistical analyses and data representation are described in the figure legends. In case data did not follow a normal distribution, a logarithmic transformation of the data was performed. A two-sided *p* < 0.05 was considered statistically significant, unless stated otherwise.*p < 0.05, **p < 0.01, ***p < 0.001,

## 3 Results

### 3.1 Isolation and flow cytometric phenotyping of decidual CD4^+^ Tregs and Tconvs

The work flow to isolate the cells of interest from maternal (m) peripheral blood (PB), umbilical cord blood (UCB) and decidua is schematically indicated in **Figure 1A**. CD4^+^ T cells purified by magnetic bead selection were characterized phenotypically by spectral flow cytometry after staining with antibodies discerning T-cell populations and cell states (**Supplementary Figure 1A**; **Supplementary Table 1**). Clustering analysis of flow cytometry data using optimized *t*-distributed Stochastic Neighbor Embedding (Opt-SNE) clearly discerned CD4^+^ T-cell populations based on tissue origin (**Figure 1B**). CD4^+^ T cells from DB and DP primarily had a CD45RO^+^ effector phenotype, while CD4^+^ T cells from mPB and UCB primarily had a naïve CD45RA^+^ phenotype (**Figure 1C**). CD4^+^ Tregs were identified by expression of FOXP3 and CD25 (IL2RA) and clustered separately from CD4^+^ Tconvs (**Figure 1C**). Effector phenotype CD45RO^+^ Tregs (FOXP3^+^) and Tconvs (FOXP3^-^) were significantly higher in frequency in DP and DB as compared to mPB and UCB (**Figure 1D**). Moreover, Tregs in DP and DB had higher expression levels of FOXP3 (**Figure 1E**), as well as Treg markers CD25 and CTLA-4 (37, 40) (**Figures 1F, G**) than Tregs in mPB. Both Tregs and Tconvs in DP and DB expressed PD-1 at higher levels than in mPB (**Figure 1H**), suggesting ongoing TCR engagement on these cells in the decidua (56).

### 3.2 scRNA sequencing of CD4^+^ Tregs and Tconvs from decidua and blood

To gain insight into the discerning and common features of CD4^+^ Tregs and Tconvs that determine human pregnancy success, we performed scRNAseq, coupled with TCRseq to clarify the clonal relationships within and between the Treg and Tconv populations. For reference, we included next to DP and DB samples, mPB and UCB samples from the same donors. CD4^+^ T-cell enriched fractions of these tissues were prepared as described in **Figure 1A**, from which Tregs and Tconvs were purified by flow cytometric sorting. Generally, CD25 and CD127 (IL7R) are used to discriminate Tregs from Tconvs respectively in cell sorting (57). However, CD127 was expressed on a proportion of decidual Tregs, disqualifying it as a selective Tconv marker (**Supplementary Figure 1B****).** On the other hand, expression of CD73, CD161 (KLRB1) and particularly CD26 (DPP4) discriminated decidual Tconvs from Tregs (**Supplementary Figures 1B, C**). Decidual CD4^+^ Tregs and Tconvs were therefore sorted on basis of reciprocal expression of CD25 and CD26, and blood CD4^+^ Tregs and Tconvs on reciprocal expression of CD25 and CD127 (**Supplementary Figures 1D, E**). After sorting, about 80% of the decidual CD4^+^ T cells expressed Treg-specific transcription factors FOXP3 and IKZF2 (37, 40) (**Supplementary Figure 1E**).

Given the low frequency of Tregs among decidual CD4^+^ T cells (**Figure 1D**), sorted Tregs and Tconvs were pooled at a 1:1 ratio per tissue sample for equal representation in the scRNAseq analysis (**Supplementary Figure 2A**). After hashtag oligonucleotide (HTO) demultiplexing and quality control, negatives, doublets and contaminating non-CD4^+^ T cells were excluded from the analysis (**Supplementary Figures 2B–E**). Of the 9861 remaining cells, 5541 cells yielded a productive *TCRA*/*TCRB* transcript pair, representing 4514 decidual and 1000 blood-derived cells (**Supplementary Figures 2F, G**) that were used for further data analysis.

**Figure 2:**
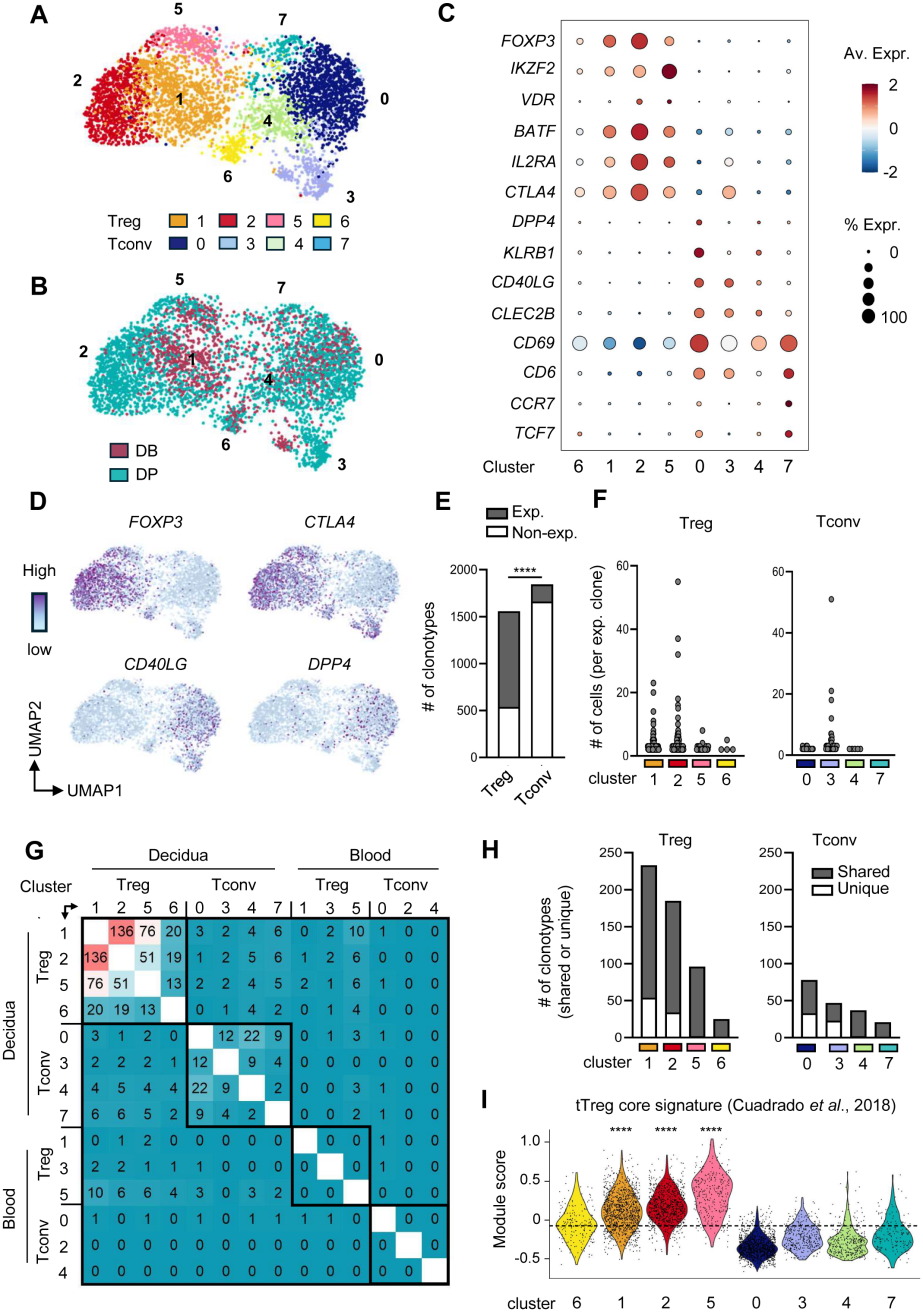
scRNAseq and TCRseq of decidual CD4+ T cells; Treg and Tconv identification. **(A, B)** UMAP projection and clustering of decidual CD4^+^ T cells (n=3, pooled, **Table 1**) based on the total transcriptome, colored by cluster **(A)** and DP or DB origin **(B)**. **(C)** Quantification of level and frequency of indicated transcripts in the Treg and Tconv clusters. *DPP4* encodes CD26. **(D)** UMAP projection of indicated transcripts in decidual CD4^+^ T cells. **(E)** Total number of Treg and Tconv clonotypes that were expanded (≥ 2 cells with the same TCR) or non-expanded (clone identified once). χ^2^-test was used for statistical analysis (****p < 0.0001). **(F)** Number (#) of expanded Treg and Tconv clonotypes per cluster. **(G)** Heatmap showing clonotype sharing between clusters in decidua and blood (see **Supplementary Figure 3**). Absolute numbers of clonotypes are indicated on the X- and Y-axes, with colors discerning degree of TCR sharing. **(H)** Number (#) of expanded Treg and Tconv clones with a TCR sequence shared between clusters, or unique for one cluster. **(I)** Violin plot depicting the module score of the tTreg core signature identified by Cuadrado et al. (44) in each decidual Treg and Tconv cluster. A one-sample *t*-test was used for statistical analysis (****p < 0.0001; dashed line represents average module score of the data set).

**Figure 3:**
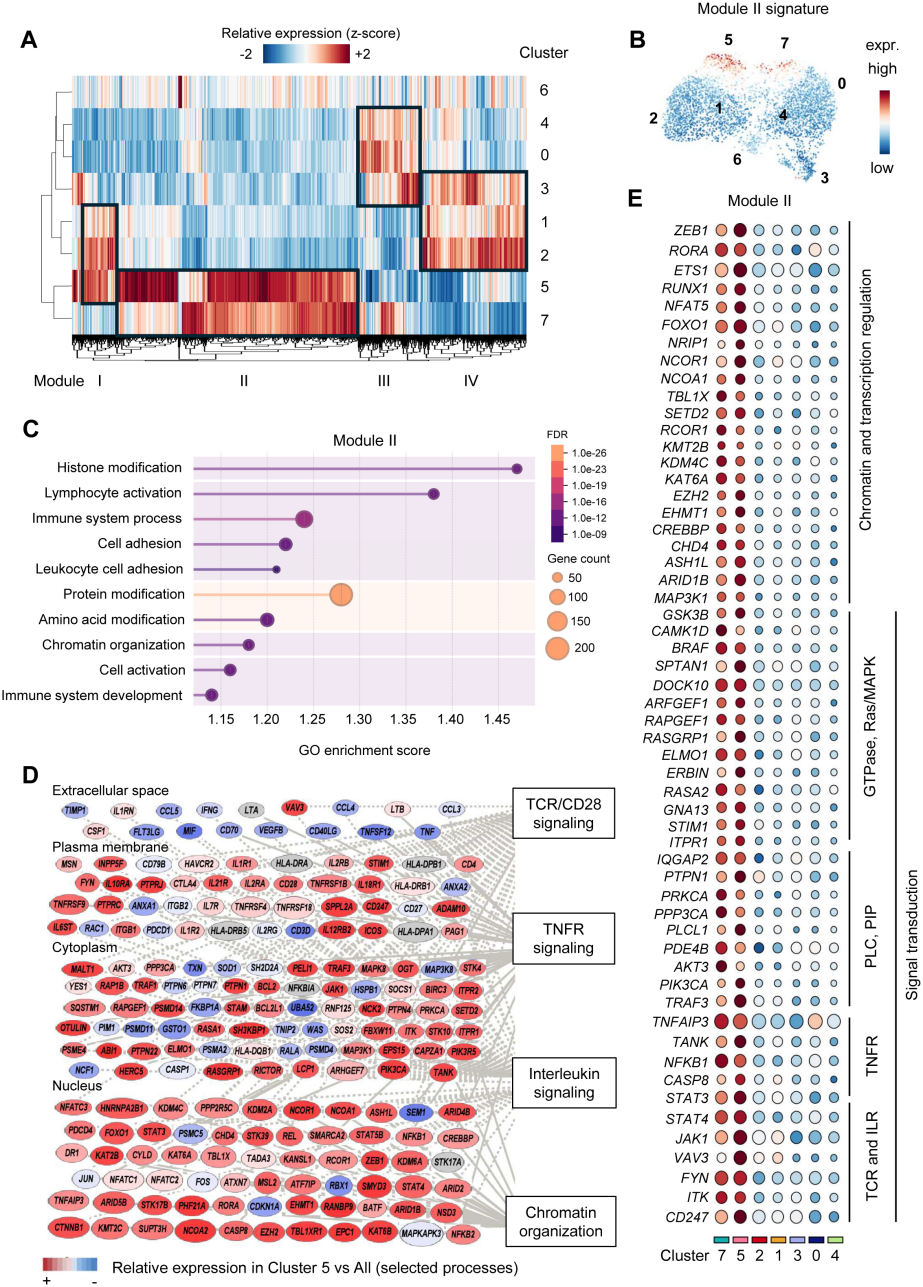
Specific decidual Treg and Tconv clusters share an activated state. **(A)** Hierarchical clustering and heatmap showing relative expression of differentially expressed genes (DEGs) for all CD4^+^ T-cell clusters identified by scRNAseq (**Supplementary Table 1**). Columns represent individual genes and rows represent the different clusters identified in **Figure 2B**. Relative expression is depicted as *z*-score for each cluster versus all other clusters. Boxes indicate gene Modules (I–IV) with similar levels of the encompassed transcripts in two or more clusters (**Supplementary Table 3**). **(B)** Module score for Module II genes shared by Treg cluster 5 and Tconv cluster 7 plotted on the UMAP projection introduced in **Figure 2A**. **(C)** Functional annotation by Gene Ontology (GO) analysis of transcripts in Module II. **(D)** DEGs associated with functional categories “TCR/CD28 signaling”, “TNFR signaling”, “interleukin signaling” and “chromatin remodeling” according to Reactome terms (**Supplementary Table 4**) depicted according to subcellular distribution by IPA. Z-scores representing transcript levels in cluster 5 relative to all other clusters were plotted. **(E)** Quantification of transcript levels and frequency of expression of indicated Module II marker genes per CD4^+^ T-cell cluster. Color legend as for **Figure 2E**.

Clustering analysis of decidual cells based on whole transcriptome data identified eight distinct cell clusters (**Figure 2A**; **Supplementary Table 2**), with partial separation of cells from DP and DB (**Figure 2B**). Cells in clusters 1, 2, 5 and 6 were identified as Tregs based on *FOXP3* expression, whereas cells in clusters 0, 3, 4 and 7 were identified as Tconvs (**Figure 2C**). Treg cluster 2 and Tconv cluster 3 were particularly enriched in DP (**Figures 3A, B**). Tconv clusters 0 and 7 showed a transcriptional profile consistent with stem-like cells that had not undergone effector differentiation, as characterized by *CCR7* and *TCF7* transcripts encoding the lymph node homing receptor CCR7 (58) and the transcription factor TCF1 (59). Treg identity of cells in clusters 1, 2 and 5 was further confirmed by elevated *IKZF2*, *IL2RA*, *CTLA4* and *BATF* transcript levels (40, 60) (**Figure 2C**). Cells in cluster 6 displayed low expression levels of these and many other transcripts. Tconv clusters 0, 3, 4 and 7 had elevated transcript levels of, amongst others, *DPP4* (encoding CD26), *KLRB1* (encoding CD161*)*, *CD40LG, CLEC2B, CD69* and *CD6* (**Figure 2C**). Representation of selected transcripts in the UMAP projection of the dataset highlighted their differential expression levels in Treg versus Tconv clusters and confirmed *FOXP3* as the optimal discriminatory marker for all Tregs (**Figure 2D**).

CD4^+^ T cells from mPB and UCB were distinct from decidual CD4^+^ T cells based on their gene expression profiles (**Supplementary Figure 2D**) and, when analyzed separately, segregated into six different clusters (**Supplementary Figures 3A, B****;** **Supplementary Table 2**). Clusters 1, 3 and 5 were annotated as Tregs based on expression of *FOXP*3, whereas clusters 0, 2 and 4 were identified as Tconvs (**Supplementary Figure 3C**). Most clusters exhibited a stem-like state, indicated by *TCF7* expression alone or combined with *CCR7*. Treg cluster 5 represented an eTreg population, lacking stem-like markers and expressing *FOXP3*, *IKFZ2, IL2RA*, *CTLA4* and *BATF* transcripts (**Supplementary Figure 3D**).

### 3.3 The decidua supports clonal expansion of tTregs as well as Tconvs

To assess clonal expansion and clonal relationships of Tregs and Tconvs in the decidua, we examined our scTCR sequencing data. Over 1500 unique T-cell clones (clonotypes) were identified for both Tregs and Tconvs based on paired *TCRA*/*TCRB* CDR3 sequences. Expanded clonotypes (clone frequency ≥ 2) were more frequent in Tregs than in Tconvs, with the highest expansion observed in Treg clusters 1 and 2 and in Tconv cluster 3 (**Figure 2E**). Most clonotypes, in both Tregs and Tconvs, were shared between clusters of the same cell lineage, rather than restricted to individual clusters (**Figure 2F**), suggesting that clusters represent distinct cell states of clonally expanded Tregs and Tconvs clones within the decidua. TCR clonotype sharing was particularly prominent among Treg clusters 1, 2, 5 and 6, and among Tconv clusters 0, 3, 4 and 7 (**Figure 2G**). In contrast, TCR clonotype sharing between Tregs and Tconvs was rare (**Figure 2G**), indicating that Tregs and Tconvs in the decidua largely derive from distinct clonal origins, rather than Tregs arising from Tconvs, which is characteristic of pTregs (36, 37, 61). These data argue that the vast majority of Tregs in the term decidua are tTregs, consistent with the low frequency TCR sharing between human decidual Tregs and Tconvs observed in recent studies (34, 35).

To further support the conclusion that decidual Tregs at term delivery are predominantly tTregs, we assessed whether they expressed the core tTreg signature defined in healthy human blood by both proteomics and transcriptomics (44). This analysis revealed significant enrichment of the tTreg core signature in decidual Treg clusters but not in Tconv clusters (**Figure 2H**). Complementary flow cytometric analysis confirmed at the protein level that Tregs in the DP expressed FOXP3, IKZF2 and IKZF4 more uniformly and at higher levels than Tregs in matched mPB (**Supplementary Figures 4A–C**). IKZF2 and IKZF4 are part of the tTreg proteomic core signature (44) and cooperate to regulate Treg signature gene expression (41, 62).

We also detected clonotype sharing between blood Treg cluster 5 and decidual Treg clusters 1, 2 and 5 (**Figure 2G**). Blood cluster 5 represented cells from both mPB and UCB (**Supplementary Figures 3A, B**) and was composed of Tregs according to *FOXP3, IKZF2, BATF, IL2RA* and *CTLA4* expression (**Supplementary Figure 3D**). While cells in blood clusters 0–4 had high levels of *IL7R, CCR7* and *TCF7* transcripts, indicative of a naïve or stem-like cell state, cells from cluster 5 had low levels of these transcripts, reflecting a more differentiated effector state (**Supplementary Figure 3D**). Although the blood clusters contained diverse clonotypes, clonotype expansion was limited relative to the decidua (**Supplementary Figures 3E, F**). Within blood, effector (e)Tregs (cluster 5) harbored the largest number of expanded clonotypes (**Supplementary Figures 3G, H**). These findings suggest that Tregs from this active and expanding cluster had migrated from mPB into the decidua.

Collectively, these data indicate that at healthy, term pregnancy, the decidua is primarily populated by tTregs rather than pTregs. Particularly tTregs, but also Tconvs undergo clonal expansion in the decidua, with most clonotypes shared between clusters of the same lineage. The differential gene expression of the same clonotypes between T cell clusters indicates that the same clones acquire distinct functional states throughout and possibly after clonal expansion.

### 3.4 Identification of actively signaling Tregs and Tconvs in the decidua

To gain insight into the functional states of the decidual Tregs and Tconvs, we performed hierarchical clustering of the DEGs that defined the decidual Treg and Tconv clusters (**Supplementary Table 2**). Based on these DEGs, we identified four gene expression modules (I–IV) representing transcriptional patterns shared between specific clusters (**Figure 3A**; **Supplementary Table 3**). Treg cluster 6 exhibited a stress-related transcriptional profile, contained few DEGs and segregated from the other Treg clusters. Consequently, it was excluded from downstream analyses.

Module II encompassed 616 genes that were similarly expressed in Treg cluster 5 and Tconv cluster 7 (**Figures 3A, B**; **Supplementary Table 3**). Gene Ontology (GO) pathway analysis identified “Lymphocyte activation” and “Histone modification” as top upregulated pathways (**Figure 3C**). It also included a number of other pathways reflecting immune cell activation, cell-cell interaction, protein modification and chromatin remodeling (**Figure 3C**). By Ingenuity Pathway Analysis (IPA), we grouped closely related Reactome pathway terms into the functional categories “TCR/CD28 signaling”, “TNFR signaling”, “interleukin signaling” and “chromatin organization” (**Supplementary Table 4**). High levels of these transcripts in Treg cluster 5 (**Figure 3D**) and Tconv cluster 7 (**Supplementary Figure 5A**) relative to other clusters illustrate the enrichment of signaling molecules and nuclear regulators within these clusters.

Depiction of transcript levels of selected, representative Module II genes across clusters (**Figure 3E**) demonstrated that Tregs and Tconvs from clusters 5 and 7 were enriched for genes associated with active signal transduction. These included components of TCR/CD28 signaling (*CD247* – encoding CD3ζ, *FYN*, *ITK* and *VAV3*), IL receptor signaling (*STAT3*, *STAT4*, *JAK1*), and TNFR signaling (*TRAF3, TANK, NFKB1, CASP8*, and *TNFAIP3*). In addition, the shared Module II included many protein kinases and phosphatases, nucleotide exchange factors for small GTPases, epigenetic regulators and transcription factors (**Figure 3E**).

This analysis suggested that Tregs and Tconvs in clusters 5 and 7, represented in both DB and DP, were in a specific state of heightened signaling activity consistent with antigen-driven activation, with TCR engagement, costimulation and cytokine input driving signal transduction and concomitant gene regulation. Cells in clusters 5 and 7 showed little or no clonal expansion, respectively (**Figure 2F**). The *CCR7^+^TCF7*^+^ stem-like state of cluster 7 cells and the *CD69* expression (63) (**Figure 2C**) are in line with an early activation and differentiation state. Based on clonotype sharing (**Figure 2G**), Tregs in cluster 5 were likely the precursors of Tregs in clusters 1 and 2.

### 3.5 Effector Tregs and Tconvs in the decidua share antigen presentation and metabolic features

**Figure 4:**
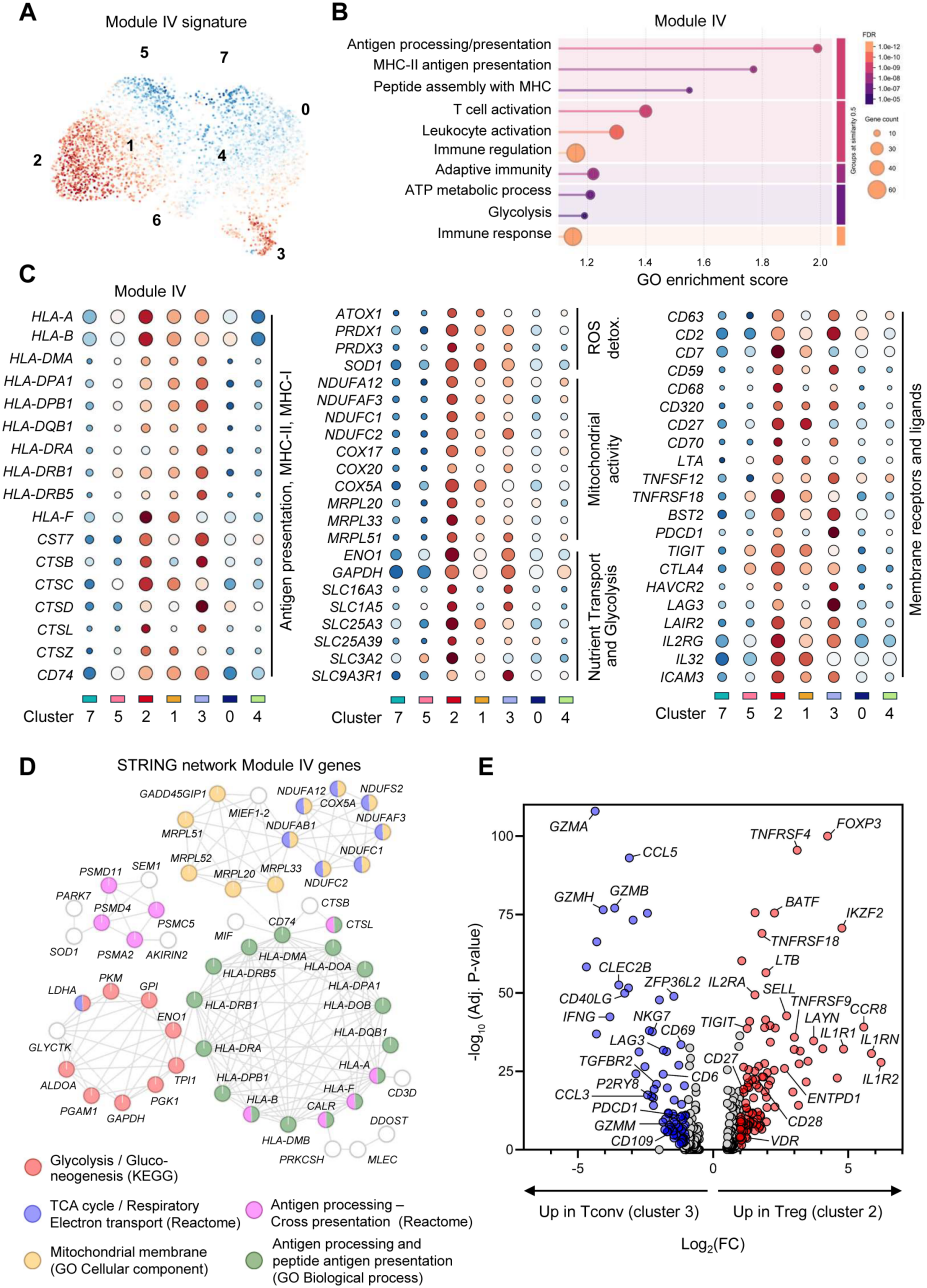
Shared and discriminating functional features of decidual Tregs and Tconvs. **(A)** Module score calculated for Module IV genes shared by Treg clusters 1, 2 and Tconv cluster 3 plotted on the UMAP projection introduced in **Figure 2A**. **(B)** Functional annotation by GO analysis of transcripts in Module IV. **(C)** Quantification of transcript levels and frequency of expression of indicated Module II marker genes per CD4^+^ T-cell cluster. Color legend as for **Figure 2E**. **(D)** Depiction of indicated functional networks of Module IV transcripts by STRING analysis. **(E)** Volcano plots showing transcripts differentially expressed between Treg cluster 2 and Tconv cluster 3.

We next focused on Module IV (**Figure 4A**), which identified additional commonalities in gene expression between Tregs and Tconvs. Module IV encompassed 263 genes shared, to varying degrees, between Treg clusters 1 and 2 and Tconv cluster 3 (**Figures 3A**, **4A**; **Supplementary Table 3**). Cells in these clusters had undergone clonal expansion, with the largest clones observed in Treg cluster 2 and Tconv cluster 3 within the DP (**Figure 2E**). Treg cluster 1 represented cells in both DB and DP and was clonally highly related to Treg cluster 2 based on TCR sharing (**Figure 2G**). The signaling and gene regulation processes that were prominently expressed in Tconv cluster 7 and Treg cluster 5 were largely silenced in Tconv cluster 3 (**Supplementary Figure 5B**) and Treg clusters 1 and 2 (**Supplementary Figures 6A, B**).

GO pathway analysis of DEGs that defined Module IV identified antigen processing and presentation by both MHC-I and MHC-II as the main functionality characterizing both Tregs and Tconvs in clusters 1, 2 and 3 (**Figure 4B**). MHC-I pathway features were indicated by upregulation of transcripts encoding *HLA-A*, -*B*, and -*F, CALR* and diverse proteasome (*PSM*) subunits (**Figures 4C, D**). Transcripts encoding a variety of MHC-II molecules (*HLA-DR*, -*DP* and DQ), as well as chaperonins *HLA*-*DM* and *CD74* (encoding the Invariant chain, Ii) were upregulated in all three clusters, along with a variety of cathepsin and cystatin (*CTS, CST*) proteases that process peptides for loading into MHC-II in late endosomes (64) (**Figures 4C, D**). HLA-DR protein expression was higher on both Tregs and Tconvs in the DP compared to mPB, with significantly higher levels on Tregs than Tconvs (**Supplementary Figure 4D**).

Tregs in clusters 1 and 2 and Tconvs in cluster 3 also shared metabolic features, specifically “glycolysis” and “ATP metabolic process” (**Figure 4D**). Accordingly, they had upregulated transcripts encoding key enzymes involved in glycolysis, including *PGK1*, *ALDOA*, *LDHA*, *ENO1, GAPDH*, as well as the lactate transporter *SLC16A3* (**Figures 4C, D**). Multiple components of respiratory chain complex I (*NDUF* family) and cytochrome c oxidase enzymes were upregulated, suggesting enhanced mitochondrial electron transport chain activity. Increased expression of reactive oxygen species (ROS) detoxifying enzymes and MRPL genes further supported elevated respiratory chain function and mitochondrial biogenesis, respectively (**Figures 4C, D**). Together, these data indicate that Tregs and Tconvs in these decidua clusters are (potentially) active effector cells with shared antigen presentation and glycolytic capacities.

### 3.6 Membrane receptors and potential effector functions of decidual Tregs and Tconvs

Genes in Module IV also encompassed transcripts encoding cell surface receptors, including the co-inhibitory receptors *CTLA4, PDCD1* (encoding PD-1)*, TIGIT, HAVCR* (encoding TIM-3)*, LAG3* and *LAIR2*, and costimulatory ligands *LTA* (encoding Lymphotoxin-α)*, TNFSF12* (encoding TWEAK) and receptors *CD27, CD70 and TNFRSF18* (encoding GITR) (**Figure 4C**; **Supplementary Table 3**). However, various molecules in this module were differentially expressed between Tregs and Tconvs. A volcano plot highlighted differential expression of characteristic surface receptors, effector molecules and transcription factors by Tregs in cluster 2 and Tconvs in cluster 3 (**Figure 4E**). This analysis clearly identified Tregs in cluster 2 as NLT-resident eTregs. These eTregs stem from tTregs (46, 48, 65) and characteristically express high levels of transcripts encoding lineage-defining transcription factors *FOXP3*, *IKZF2*, *BATF*, costimulatory receptors *TNFRSF4* (OX40), *TNFRSF9* (4-1BB), *TNFRSF18* (GITR), coinhibitory receptor *TIGIT*, chemokine receptor *CCR8*, adhesion receptor *LAYN* and ATP/ADP hydrolyzing enzyme *ENTPD1* (CD39) (33, 45, 46, 48, 65, 66). These proteins were indeed expressed at higher levels at the cell surface by Tregs than by Tconvs in the DP. Also, CTLA4 protein levels were higher in Tregs than in Tconvs in the DP (**Supplementary Figure 4D**). High expression of CTLA4 and CD39 suggests the exertion of defined suppressive functions by decidual eTregs, namely attenuation of CD28 costimulation (67) and conversion of ATP into immunosuppressive adenosine (68), respectively. Tconvs in cluster 3 were set apart from Tregs in cluster 2 particularly by the higher levels of transcripts encoding cytotoxic effector molecules, including Granzymes (*GZMA, GZMB, GZMH, GZMM*) and IFNγ (*IFNG*) (**Figure 4E**; **Supplementary Table 2**).

**Figure 5:**
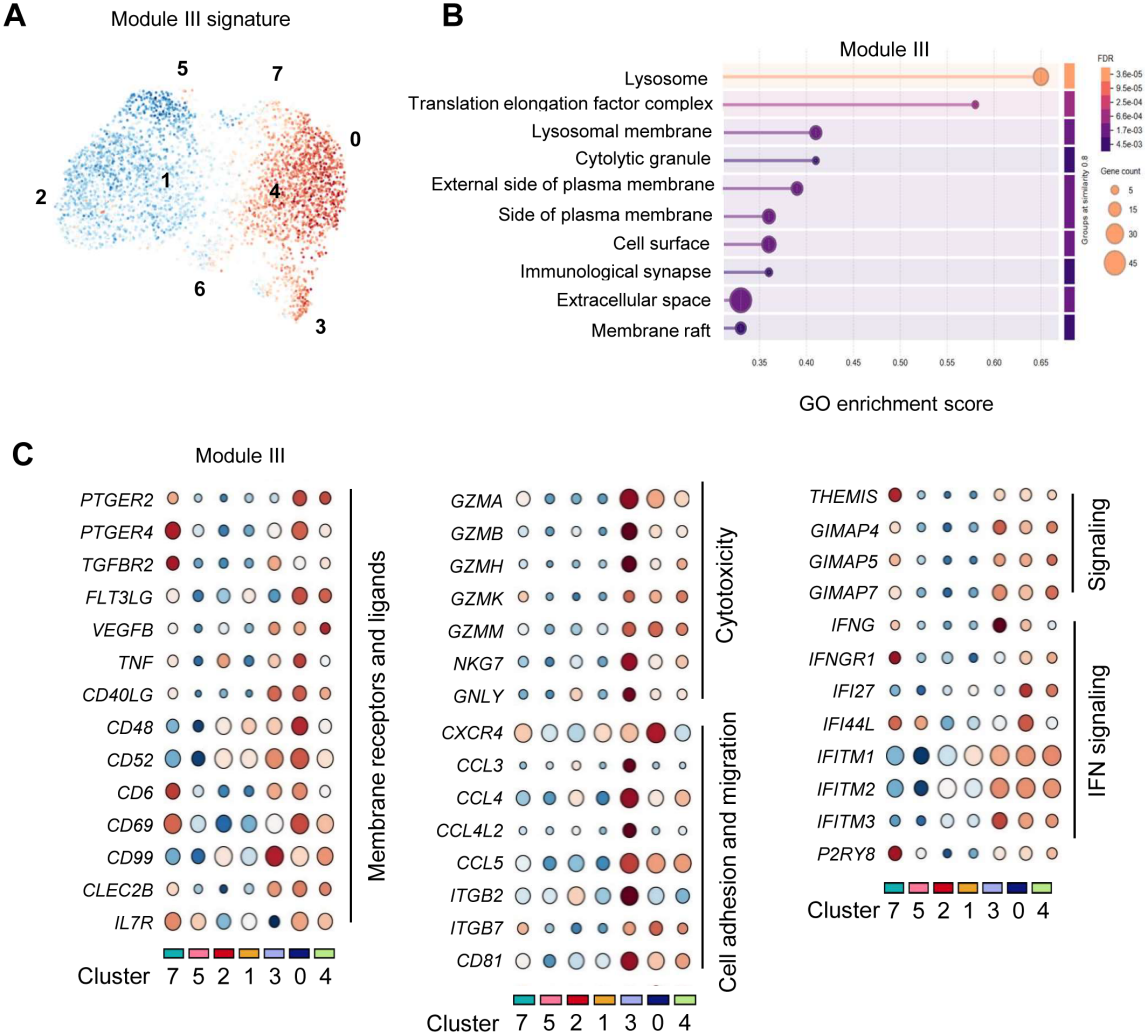
Analysis of Tconv-specific Module III transcripts. **(A)** Module score calculated for Tconv-specific Module III genes plotted on the UMAP projection of the decidual T-cell clusters introduced in **Figure 2A**. **(B)** Functional annotation by GO analysis of transcripts in Module III. **(C)** Quantification of transcript levels and frequency of expression of indicated Module III marker genes per CD4^+^ T-cell cluster. Color legend as for **Figure 2E**.

Module III comprised Tconv-specific transcripts in clusters 0, 3 and 4, which were shared in part with Tconv cluster 7 (**Figures 3A**, **5A**). GO analysis of Module III genes revealed that Tconvs in clusters 0, 3 and 4 contained cytotoxic granules, which are lysosomal structures (**Figure 5B**). Indeed, Module III included many cytotoxic enzymes that reside in such granules (**Figure 5C**). Module III also suggested autocrine IFN signaling, based on expression of *IFNG* and *IFNGR1*, and various IFN response (*IFI*) genes. Transcripts further suggested production of TNF, FLT3L and VEGFB and chemokines CCL4 and CCL5 by decidual Tconvs (**Figure 5C**). Expression of chemokines, chemokine receptors and specific integrins suggested migration and cell-cell communication. Among cell surface receptors in this module, transcripts encoding CLEC2B, CD40LG, CD6, CD26 (*DPP4*) and KLRB1 distinguished Tconvs from Tregs, as seen before, as did transcripts encoding TGFβ receptor-2 (TGFBR2), prostaglandin receptors-2 and -4 (*PTGR2, PTGR4*) and various other molecules (**Figure 5C**; **Supplementary Table 3**).

Together, these data highlight key phenotypic distinctions between decidual Tregs and Tconvs and suggest that decidual eTregs may exert suppressive functions, while CD4^+^ eTconvs have cytolytic and pro-inflammatory potential. Effector Tregs and Tconvs display antigen presenting capacity via both MHC-I and MHC-II, suggesting that they may engage in antigen-specific, synaptic communication with each other, both between and within cell lineages. Through these interactions, Tregs may specifically suppress proinflammatory and cytotoxic Tconv functions *in situ*.

### 3.7 Changes accompanying the transition of decidual Tregs from the signaling to the effector state

**Figure 6:**
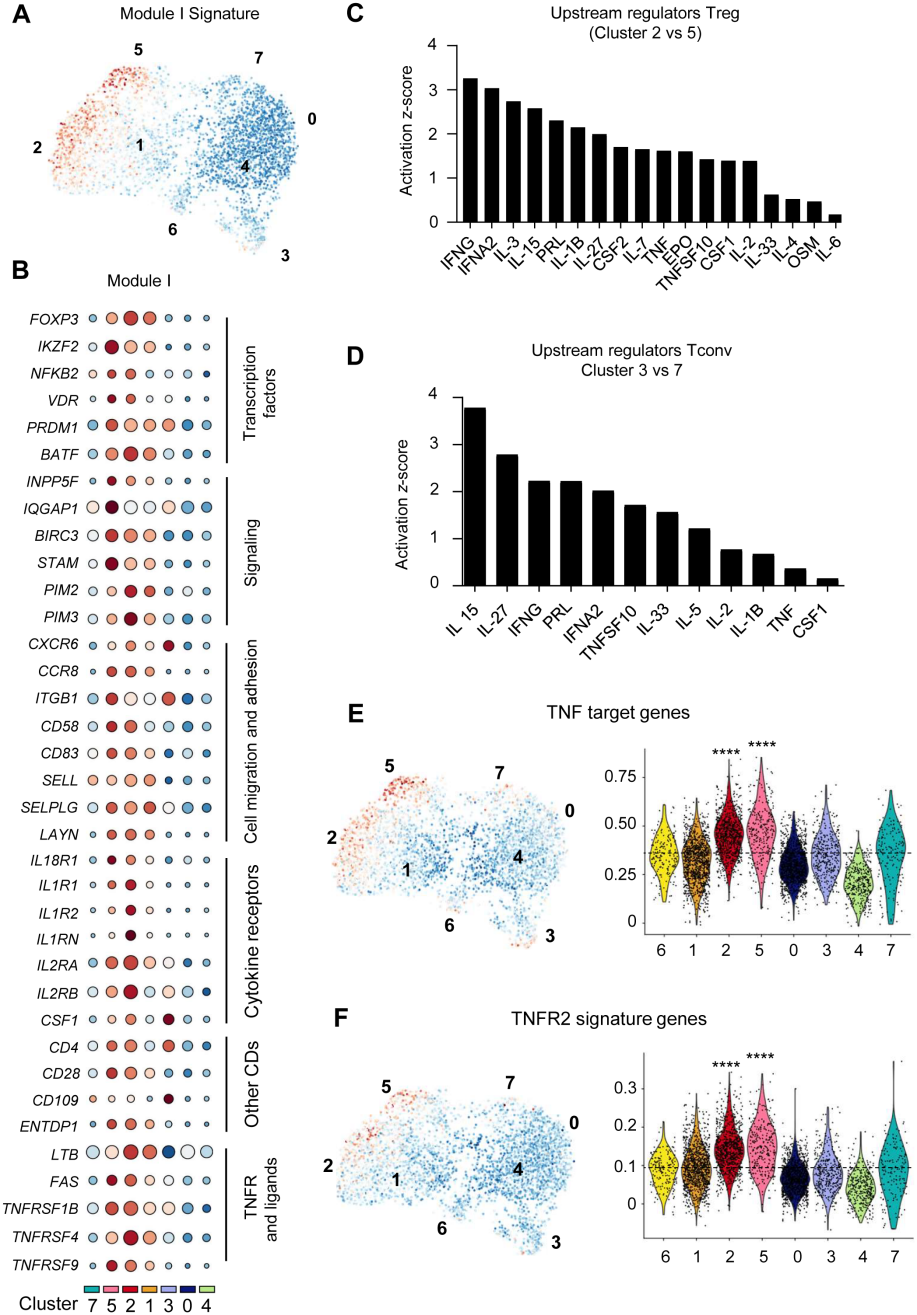
Drivers of Treg and Tconv differentiation in the decidua. **(A)** Module score calculated for Treg-specific Module I genes plotted on the UMAP projection of the decidual T-cell clusters introduced in **Figure 2A**. **(B)** Quantification of transcript levels and frequency of expression of indicated Module I marker genes per CD4^+^ T-cell cluster. Color legend as for **Figure 2E**. **(C, D)** Upstream Regulator Analysis in IPA was performed using relative expression values (*z*-scores; cut-off value < -0.1 or > 0.1) of DEGs from Treg cluster 2 versus 5 **(C)**, and Tconv cluster 3 versus 7 **(D)**. Upstream regulators were filtered on categories cytokines and growth factors. Depicted are positive upstream regulators (cut-off value of *p* < 1e-10). **(E, F)** Module scores calculated for TNF target genes identified by IPA **(E)** and the gene signature of TNFR2 costimulated tTregs (logFC>0.5) identified by Mensink et al. (48) **(F)** plotted on the UMAP projection introduced in **Figure 2A** (left), and visualized in a Violin plot (right). A one-sample t-test was used for statistical analysis, ****p<0.0001; dashed line represents average module score of the data set.

Module I encompassed 109 genes that were primarily expressed in decidual Tregs and partially shared between the actively signaling Treg cluster 5 and the more differentiated, effector type Treg clusters 2 and 1 (**Figures 3A**, **6A**). Treg cluster 5 expressed Module I transcripts next to Module II transcripts that reflect an activated state, while Treg clusters 2 and 1 expressed Module I transcripts alongside Module IV, indicative of the eTreg differentiation state (**Figure 3A**). These data indicate that Module I transcripts were likely upregulated in the transition from the cluster 5 cell state to the cluster 2 and 1 cell states. Pathway analysis of Module I genes highlighted cytokine signaling, including TNF- and NF-κB signaling in these Treg clusters, as well as T-cell differentiation (**Supplementary Figure 7A**). Module I contained *TNFRSF1B* (encoding TNFR2) and *NFKB2* transcripts (**Figure 6B**) and in agreement, TNFR2 protein was expressed at much higher level on Tregs than on Tconvs in the DP (**Supplementary Figure 4D**). Module I also included *TNFRSF4*, *TNFRSF9* and FAS (*TNFRSF6*) transcripts (**Figure 6B**), all of which showed higher cell-surface protein expression on Tregs than on Tconvs within the DP (**Supplementary Figure 4D**).

Module I further included *FOXP3*, *IKZF2* and other transcription factors characteristic for (effector) Tregs, including *BATF*, *PRDM1* (encoding Blimp-1) and *VDR* (encoding the Vitamin D receptor) (**Figure 6B**). Upregulation of these markers, together with *CCR8*, *ENTPD1* and *LAYN* supports ongoing or completed differentiation of tTregs into NLT-resident eTregs in clusters 1 and 2 (**Figure 6B**). *CD28*, *IL2RA*, and *IL2RB* upregulation aligns with the observed clonal expansion in clusters 1 and 2, as CD28 costimulation enhances TCR-driven activation, cell cycle entry and survival (69), while IL-2 receptor signaling in Tregs upon stimulation by Tconv-derived IL-2 is critical for Treg proliferation (42).

A striking upregulation of transcripts encoding IL-1 and IL-18 receptors, as well as IL-1 antagonist IL1RN (**Figure 6B**), suggests a potentially transient role for these cytokines, which, similar to TNFRs, activate NF-κB and MAPK pathways. The collective data support the concept that stem-like tTregs undergo activation (cluster 5, Module II), clonal expansion and concomitant progressive differentiation into NLT-resident eTregs within the decidua (clusters 1 and 2, Module I), with the most expanded, effector phenotype Tregs being enriched in the DP (cluster 2, **Figure 2E**).

### 3.8 Drivers of Treg and Tconv effector differentiation in the decidua

Like tTregs, Tconvs also underwent clonal expansion and effector differentiation in the decidua, as suggested by the stem-like and early activation transcriptional profiles of cluster 7 cells (Module II) and the (cytotoxic) effector state of cells in clusters 0, 4 and 3 (Module III). Cluster 3 contained the most expanded clones (**Figure 2E**) and resided in the DP (**Figures 2A, B**), suggesting that this cluster represents the most differentiated state of decidual Tconvs. To identify factors that potentially drove the differentiation of decidual Tregs and Tconvs, we performed upstream regulator analysis using IPA and compared the stem-like clusters with the differentiated and most expanded clusters (**Figures 6C, D**). Several growth and differentiation factors were implicated in regulating gene expression in both decidual Tregs and Tconvs, including type I and type II IFNs, IL-2, IL-1B, other IL molecules, and Prolactin (PRL), whose receptors all regulate transcription via JAK-STAT pathways. IFNγ and IL-2 response genes were most prominently expressed in Treg clusters 5 and 2, and in a proportion of the cells in Treg cluster 1 (**Supplementary Figure 7B**), consistent with these factors driving Treg expansion and differentiation towards the cluster 2 state. Among Tconvs, cluster 3 cells primarily exhibited the signature of these upstream regulators, while IL-1B signaling signatures were observed across most decidual Tregs and Tconvs (**Supplementary Figure 7B**).

TNF targets were enriched particularly in Treg clusters 5 and 2, and to a lesser extent in Tconv clusters 7 and 3 (**Figure 6E**). We recently identified TNFR2 costimulation as an important driver of naive human tTreg differentiation into NLT-resident eTregs (48). Specific upregulation of TNFR2 (*TNFRSF1B*) in Tregs in Module I (**Figure 6B**) is consistent with TNFR2 costimulation contributing to the differentiation process from the Treg cluster 5 to the Treg cluster 2 state. Module scoring based on the TNFR2-driven gene expression signature as defined in human effector tTregs by Mensink et al. (48) (**Supplementary Table 5**) supported that TNFR2 costimulation of Treg cluster 5 cells promotes their differentiation into NLT-resident eTregs in the decidua (**Figure 6F**). The TNFR2 signature was not found in any of the Tconv clusters, in line with lower TNFR2 (TNFRSF1B) protein expression on Tconvs in both decidua and blood (**Supplementary Figure 4D**), and with the selective impact of TNFR2 costimulation on human Tregs as opposed Tconvs (48, 70). These data argue that decidual tTregs expand and differentiate in the human decidua upon TCR-mediated antigen recognition and costimulation by a TNFR2 ligand, possibly TNF.

### 3.9 Comparison of decidual T cells to T cells in the tumor micro-environment

**Figure 7:**
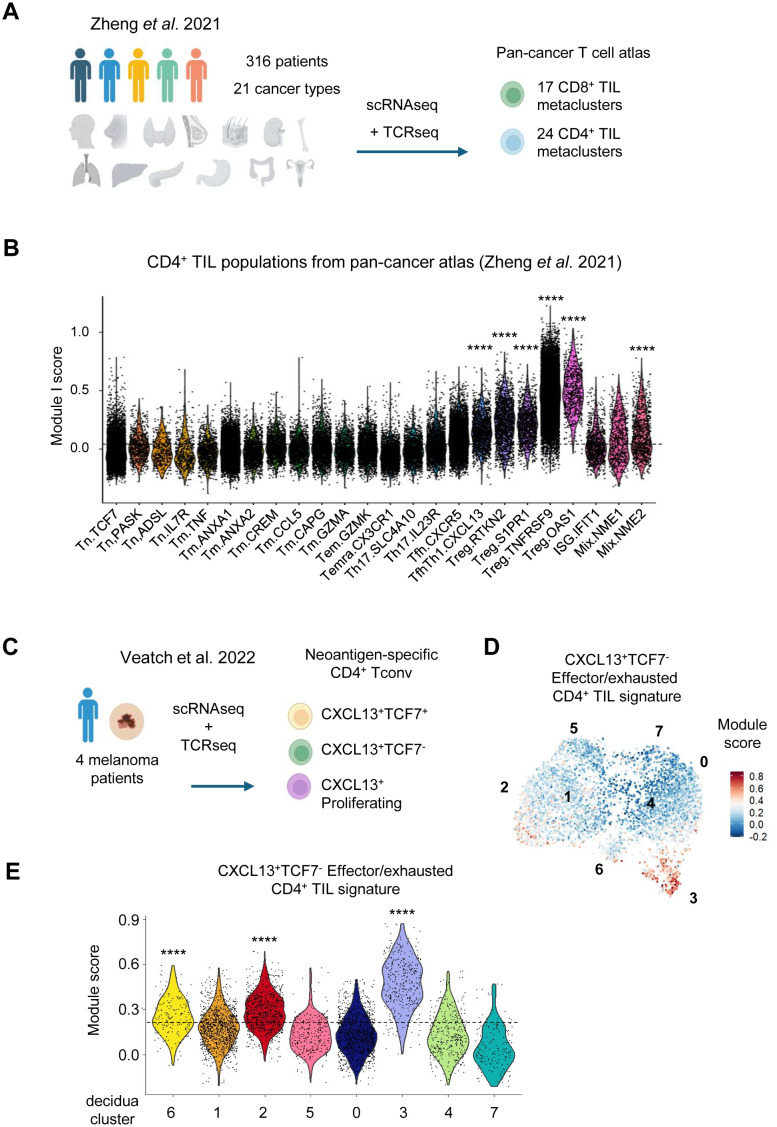
Transcriptome comparisons of decidual and tumor-resident CD4^+^ T cells. **(A)** Outline of experimental conditions that generated the scRNAseq pan-cancer atlas from Zheng et al. (55) containing expression profiles of CD8^+^ and CD4^+^ tumor-infiltrating lymphocytes (TILs) from 21 different cancer types. Created in BioRender (https://BioRender.com/igjoikd). **(B)** Violin plot showing enrichment of Module I transcripts of decidual Tregs (**Figures 4A**, **7B**; **Supplementary Table 3**) in the different CD4^+^ T-cell clusters identified in cancer by Zheng et al. (55). **(C)** Outline of experimental conditions that generated the scRNAseq dataset of Veatch et al. (54), containing expression profiles of three subsets of tumor-infiltrating, neoantigen-specific CD4^+^ Tconvs from 4 melanoma patients. Created in BioRender (https://BioRender.com/hyn2qow). **(D)** Module score of the top 100 marker genes for CXCL13^+^TCF7^-^ exhausted/effector CD4^+^ TILs from Veatch et al. (54) plotted on the UMAP projection of decidual CD4^+^ T-cell transcriptome data introduced in **Figure 2A**. **(E)** Violin plot showing the module score calculated for CXCL13^+^TCF7^-^ exhausted/effector CD4^+^ TIL signature identified by Veatch et al. (54) per cluster for indicated decidual Treg and Tconv clusters. **(B, E)** A one-sample *t*-test was used for statistical analysis (****p < 0.0001; dashed line represents average module score of the data set).

To further characterize the phenotypes observed for Tregs and Tconvs in the decidua, we performed comparative transcriptomic analyses with human cancer datasets. Firstly, we compared the Module I signature of decidual Tregs (**Supplementary Table 3**) to scRNAseq data from Zheng et al. (55), who published a pan-cancer atlas of CD4^+^ and CD8^+^ T cells across 21 different cancer types (**Figure 7A**). High module score for Module I genes in the Treg.TNFSF9 and Treg.OAS clusters (**Figure 7B**) indicated transcriptional similarity between decidual Tregs and tumor-infiltrating Tregs across diverse human cancers.

The transcriptomes of decidual Tregs and Tconvs were also compared to data from Veatch et al. (54). These authors characterized melanoma-infiltrating CD4^+^ Tconvs by scRNAseq and TCRseq, defining three populations of tumor neoantigen-specific CD4^+^ Tconvs with a CXCL13^+^ phenotype, including TCF7^+^ memory-like, TCF7^-^ effector/exhausted and proliferating cells (**Figure 7C**). The signature of CXCL13^+^TCF7^-^ effector/exhausted tumor-specific CD4^+^ Tconvs (**Supplementary Table 5**) was enriched in decidual Tconv cluster 3 and, to a lesser extent, in decidual Treg cluster 2 (**Figures 7D, E**). These data align with the clonal expansion and differentiation states of cells in these clusters, arguing that continuous TCR signaling, in conjunction with other stimuli (**Figures 6C, D**) promotes effector differentiation and features of “exhaustion” as defined in chronic infection and cancer (71).

## 4 Discussion

We here present the gene expression profiles of CD4^+^ Tregs and Tconvs in the human decidua at term delivery by C-section. Our data 1) define the great majority of decidual Tregs as tTregs; 2) highlight many discerning features of decidual tTregs and CD4^+^ Tconvs; 3) elucidate (dormant) functionalities of decidual tTregs and CD4^+^ Tconvs; 4) uncover local activation, clonal expansion and effector differentiation of tTregs and CD4^+^ Tconvs in the decidua and 5) identify upstream drivers of these processes. Transcriptomics was done on CD4^+^ T cells from three different donors to capture possible biological variability. These cells were pooled to obtain sufficient numbers for robust Treg and Tconv sorting and transcriptome analysis. Since the donors had comparable physiological states (**Table 1**), an n=3 pool likely provided a representative view of the cell types and cell states, also given that our findings align with those by Li et al. (35) and Mensink et al (48). Our scRNAseq dataset provides a foundation for future studies, including examination of suggested novel surface markers to discern NLT-resident tTregs from CD4^+^ Tconvs. Validation is required because mRNA and protein expression are only about 75% concordant due to post-transcriptional regulation. Moreover, overall protein expression does not equal cell surface expression (44). We found both by transcriptomics and flow cytometry that CD127, a cell surface marker generally used to discern CD4^+^ Tconvs from Tregs (57), is not a discriminating marker for NLT-resident cells of these two lineages, in line with earlier observations (72). Instead, it was found here and in an earlier study (73) that CD26 (DPP4) is a useful marker, given its specific expression at both mRNA and cell surface protein level on CD4^+^ Tconvs.

We prove that the dominant human *FOXP3^+^* populations at term delivery in both DB and DP are tTregs, since they are clonally distinct from Tconvs and express the tTreg core transcriptional program (44). In agreement, recent studies reported that the great majority of human Tregs in healthy first trimester total decidua (35), as well as first trimester and term DB (34) are clonally distinct from decidual Tconvs. A minority of decidual Tregs shared TCRs with Tconvs in all three studies, suggesting the additional presence of pTregs. TCR sharing between Tregs in decidua and maternal blood in the first trimester (35) supports the concept from mouse studies that T-cell responses to the fetus are primed in secondary lymphoid organs (SLOs) of the mother. Interestingly, we find that decidual Tregs still share TCRs with Tregs in maternal blood at term, suggesting that Tregs in the decidua are continuously replenished from the maternal blood. The antigen specificity of Tregs responding in pregnancy is currently unknown, but the fact that they are tTregs suggests reactivity to self-antigens (42). This is a new viewpoint on the specificity of decidual Tregs that are generally thought to respond to alloantigens. Moreover, Tregs are often depicted to interact with fetal trophoblasts in an antigen-specific manner, *e.g* (74), but this is unlikely since trophoblasts do not express MHC-II molecules (6, 7), in contrast to maternal immune cells.

The current findings argue that in human and likely also in murine pregnancy, tTregs are continuously primed on professional antigen-presenting cells in SLOs and migrate to the decidua where they are re-activated. They then locally expand and undergo (further) effector differentiation, followed by exhaustion and likely cell death, necessitating a dynamic replacement by newly primed cells. The possibility is still open that in the second trimester pTregs are more abundant than in the first trimester and at term. A role for pTregs in pregnancy success was deduced from increased fetal loss in mice that were genetically deficient in the regulatory element of the *Foxp3* gene locus that is responsible for pTreg conversion, as compared to wild-type mice (32). However, the pTreg-deficient mice were not compared in the same study to mice that lacked both pTregs and tTregs, leaving open the possibility that tTregs make a larger contribution to maternal-fetal tolerance and successful pregnancy. Accordingly, antibody-based depletion of CCR8^+^ Tregs from the decidua in mice resulted in pregnancy failure, and fetal loss in abortion-prone mice could be rescued by adoptive transfer of CCR8^+^ Tregs (35). These experiments were prompted by the observation that CCR8^+^ Tregs predominate in the human first trimester decidua.

CCR8 is one of the key markers of NLT-resident Tregs (45–48) and here we classify human decidual effector-phenotype Tregs as NLT-resident Tregs. These are tTregs that upon priming in SLO (65) differentiate under influence of BATF and IRF4 (60, 75) into effector cells with high expression of tTreg core signature genes (44), as well as pan-NLT signature genes (45–47). NLT-resident Tregs are recognizable by expression of CCR8, LAYN, ENTPD1 (CD39), TIGIT, a variety of TNFR family members, elements of TNF/NF-κB signaling and molecules involved in tissue regeneration (45–48). Accordingly, decidual Tregs expressed, as compared to blood Tregs, increased protein expression of FOXP3, CTLA-4, CD25, OX40 (encoded by *TNFRSF4*), 4-1BB (encoded by *TNFRSF9*), GITR (encoded by *TNFRSF18*), TIGIT, PD-1 and ICOS as shown by us (48) and others (33, 35). It is now clear that NLTs are generally populated by tTregs, except for the gut, where pTregs are prevalent (76). Burton et al. (47) demonstrated in mice by parabiosis that these Tregs are not primed by tissue-specific antigens but rather can enter and be maintained in any NLT, where they are replaced by new cells entering from dLNs within about three weeks. This scenario explains why we still see recently (re)activated CD4^+^ Tregs in the decidua at term pregnancy. The same scenario of dynamic turnover holds for CD4^+^ Tconvs according to our data. Thus, the decidua is populated with tTregs that most likely recognize ubiquitous self-antigens, but restrict responses of Tconvs to non-self antigens. Self-antigen specificity of NLT-resident Tregs suits their role in maintaining immunological tolerance (77) and promoting tissue regeneration under homeostasis and as well as immune challenge (78, 79).

In our study, cluster 5 Tregs and cluster 7 Tconvs shared the highly activated state. Gene expression and TCR analysis argued that Tregs and Tconvs clonally expanded and underwent effector differentiation from this state onwards, suggesting local antigen recognition by both Tregs and Tconvs. Possibly, T cells contact cDCs or other antigen-presenting cells, particularly monocyte-derived DCs and/or macrophages (80, 81) in the decidua to reinforce signals acquired during priming in SLOs. The expression of PD-1 and other tumor-derived “exhaustion” signature genes in cells of Treg cluster 2 and Tconv cluster 3 that contained the most expanded clones argue for continued TCR stimulation in the decidua. The frequency of clonally expanded populations of decidual eTregs is higher in the third trimester than in the first (34), supporting that T-cell responses to antigen in the decidua are ongoing during fetal growth.

On basis of the current data, we propose to redefine decidual Treg populations previously identified on cell surface markers and *in vitro* suppressive activity (49). Of these, the CD25^high^ population expressed both FOXP3 and IKZF2 (HELIOS) and qualifies as the eTreg population found by us and others (33, 35). The TIGIT^high^ population may be equivalent to our cluster 5 of activated Tregs, based on the markers reported. The PD-1^high^ population lacked core Treg markers and is most likely equivalent to our cluster 3 of eTconvs, also based *IFNG* expression. Several molecules expressed on decidual eTregs have well-documented suppressive roles, in particular CTLA-4 (82). CTLA-4 acts in *trans* to downregulate CD80 and CD86 by transendocytosis, which attenuates the costimulatory capacity of cDCs and T cells (67). ENTPD1 (CD39) suppresses eTconv activity by promoting conversion of ATP in immunosuppressive adenosine (68). By means of the IL-2 receptor, Tregs can deprive Tconvs of IL-2 to limit their expansion and survival (83). Additionally, the IL-1 antagonist IL1RN identified here, exerts an anti-inflammatory effect on Tconvs (84). Furthermore, TIGIT was shown to have suppressive activity *in trans (*85). Coinhibitory receptors other than CTLA-4 and TIGIT, such as PD-1 act *in cis*, restraining the activity of the cells on which they are expressed (86). Anti-inflammatory IL-10 that is in the pan-NLT eTreg signature (45) was found in decidual Tregs by others (33, 35) but not by us. At present, the mechanisms of suppression employed by NLT-resident eTregs *in situ*, including those in the decidua, remain to be addressed by specific genetic interference.

The CD4^+^ eTconvs in cluster 3 were set apart from eTregs by their proinflammatory and cytotoxic state. These eTconvs were predicted to have cytotoxic granules that can be mobilized to kill target cells by means of Granzymes and Perforin (PRF), which was indeed expressed at mRNA level in cluster 3 (**Supplementary Table 2**). Li et al. (35) also found CD4^+^ Tconvs expressing transcripts encoding Granzymes and Perforin in first trimester decidua. Effector phenotype CD8^+^ T cells in the human decidua had low protein levels of Perforin as compared to effector CD8^+^ T cells from peripheral blood, suggesting attenuation of cytotoxicity. However, these cells could degranulate and produce proinflammatory cytokines upon re-activation *in vitro (*87–89). Likely, the same holds for decidual effector CD4^+^ Tconvs. Interestingly, we found *ZFP36L2* expression in cluster 3 CD4^+^ Tconvs (**Figure 5E**), which may attenuate their proinflammatory state, since ZFP36L proteins suppress production of proinflammatory cytokines by promoting mRNA decay (90). Altogether, these data suggest that decidual eTconvs are in an alert state with proinflammatory and cytotoxic potential that may be kept in check by their own coinhibitory receptors, ZFP36L2 and Tregs, but can be mobilized *e.g.* during infection (91). Li et al. (35) found *IFNG* mRNA expression by decidual Tconv cells in the first trimester, and we identified IFNs as potential upstream drivers of both Tconv and Treg effector differentiation at term (**Figures 7C, D**). These findings are in line with inflammation being important at these stages, for placentation and delivery (91). It will be of interest to know whether the IFN signaling imprint is also present in the second trimester, which is reportedly more dominated by Th2-type cytokines (91). Importantly, tTregs maintain their identity in pro-inflammatory environments (44), so continuous exposure to IFNs does not interfere with their suppressive function.

Decidual eTregs and eTconvs expressed many components of both the MHC-II (HLA-DR, -DP, -DQ) and MHC-I (HLA-A, -B, -F) antigen presentation pathways. This intriguing finding suggests that Tregs and Tconvs may present antigens to each other, at least in MHC-II, to enable coordinated responses upon antigen-driven synaptic communication. MHC-II resides in endolysosomes and presents molecules from the local environment. MHC-I presents endogenous antigens, while in cDCs (64, 92) and perhaps also in decidual CD4^+^ Tregs and Tconvs, it can also crosspresent exogenous antigens. Possibly, presentation of endogenous antigens in MHC-I serves local monitoring of the CD4^+^ Treg and Tconv cell states by decidual CD8^+^ T cells and their elimination upon infection. The function of HLA-F, which in its non-peptide bound form is a ligand for NK cell receptor KIR3DS1 (93), is understudied and merits attention.

We identified TNF as potential upstream driver for decidual Tregs and Tconvs, with a higher confidence for Tregs (**Figures 7C, D**). *TNFR2* (*TNFRSF1B*) transcript was upregulated in decidual Tregs at the early activation state (cluster 2) and protein levels were high in Tregs, but not Tconvs (**Supplementary Figure 6**). We previously found that TNFR2 costimulation drives differentiation of human tTregs into NLT-resident Tregs *in vitro (*48) and we now identified our defined TNFR2-driven gene signature in decidual Tregs. These data strongly support a role for TNFR2 in decidual eTreg differentiation *in vivo*. We also found that that costimulation via TNFR2, but not CD28 induces a glycolytic state in CD3-activated tTregs *in vitro (*70). The decidual eTregs were indeed glycolytic, in accordance with TNFR2 input *in vivo*. TNFR2 selectively impacts on effector differentiation of Tregs and not Tconvs (48), as also observed here. In mouse models, TNFR2 endowed Tregs with stability and suppressive function in inflammatory environments (94–96), in line with current findings. It is not known whether TNFR2 costimulation of Tregs *in vivo* is induced by TNF or by its other ligands lymphotoxin α3 (LTα3) and LTα2β1 (97). *LTA* and *LTB* transcripts were expressed in decidual Tregs (**Figure 5C**, **7B**) and present in the transcriptomic signature of NLT-resident Tregs (48), suggesting that these cells may signal via TNFR2 in an autocrine fashion (98). Other potential drivers of decidual Treg and Tconv effector differentiation included Prolactin (PRL), a pituitary hormone that stimulates milk production. Interestingly, the decidua also makes PRL, where it regulates placental morphogenesis and modulates inflammation (99). Altogether, the identification of decidual Tregs as NLT-resident Tregs supports their role in attenuating inflammatory Tconv responses, but also highlights the potential of Treg cells and other immune cells to guide morphogenesis, which is highly relevant at the maternal-fetal interface.

## Data Availability

The scRNAseq and TCRseq data generated for this study have been deposited in the GEO database under accession number GSE339128.
