# Supplementary material for: Defining nature, responses and drivers of regulatory- and conventional CD4+ T cells in the human term decidua
Source: Front Immunol. 2026 Aug 4;17:1865340. doi: 10.3389/fimmu.2026.1865340 (PMC13481866; doi:10.3389/fimmu.2026.1865340)
Supplement: Supplementary file 1 [file DataSheet1.pdf]

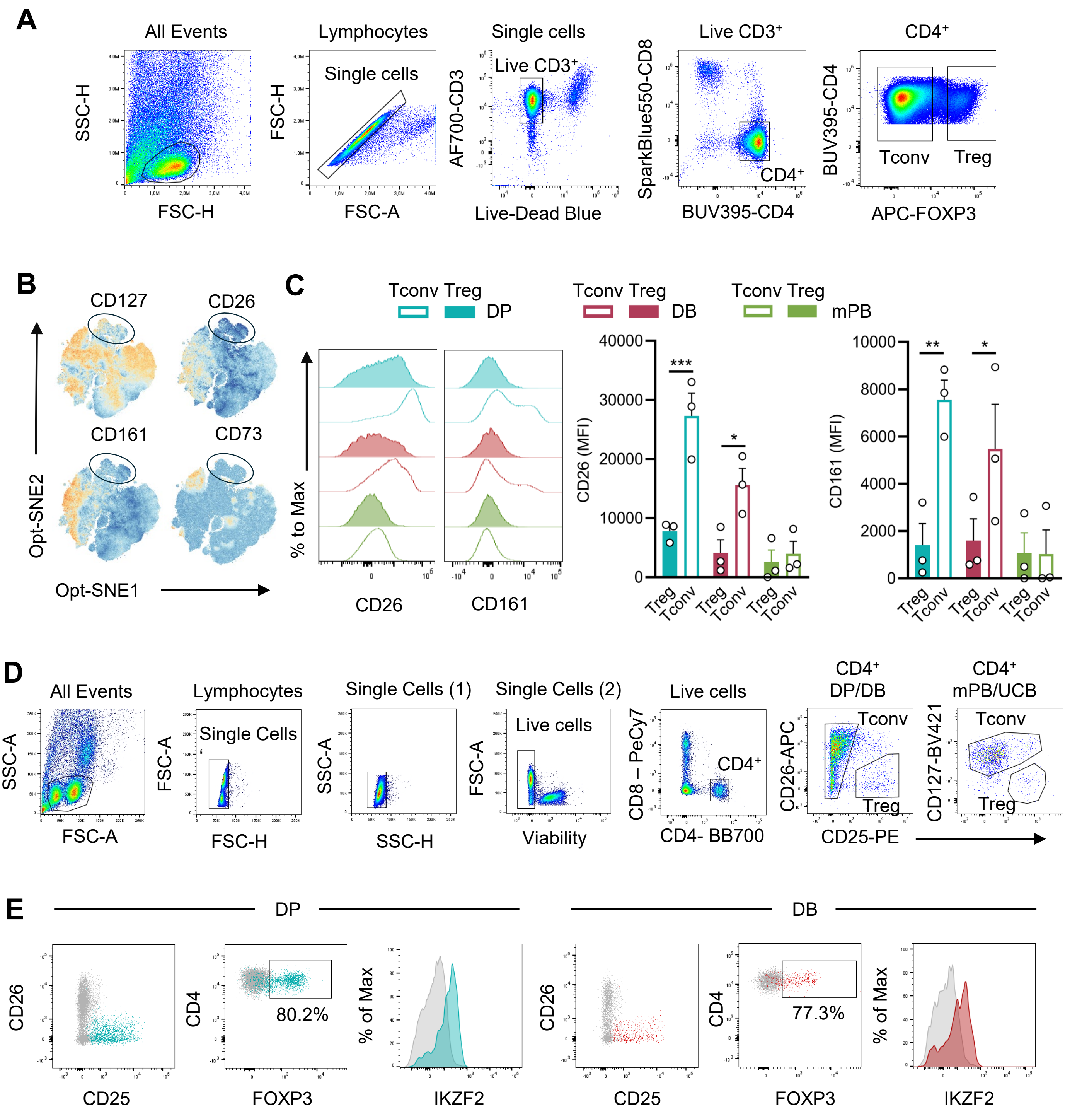

Supplementary Figure 1. Gating strategies for phenotypic analysis and cell sorting by flow cytometry. (**A**) Gating strategy used to identify Tregs and Tconvs in the CD4⁺ enriched fraction of decidua and blood cells isolated as described in Figure 1A**.** (**B**) Opt-SNE cluster analysis of concatenated flow cytometric data from all CD4⁺ T-cell samples, indicating relative expression of indicated markers. FOXP3⁺ cells (as described in Figure 1C) are encircled/highlighted. (**C**) CD26 and CD161 expression on CD4⁺ Tconvs and Tregs from indicated tissues. *Left panel*: representative histograms; *right panel*: quantification of mean fluorescence intensity (MFI). Two-way repeated measures ANOVA with Bonferroni's post hoc test was used for statistical analysis (**p*<0.05, ***p*<0.01, ****p*<0.001). (**D**) Strategies used for flow cytometric sorting of CD4⁺ Tregs and Tconvs from DP and DB on CD25 and CD26 and from mPB and UCB on CD25 and CD127, to generate samples for scRNAseq and coupled TCRseq. (**E**) Validation by flow cytometry of decidual Treg sorting on basis of CD25 and CD26 markers, as assessed by FOXP3 and IKZF2 expression. (**A-E**) Data represent samples from *n*=3 independent donors, see Table 1. (**C**) Data are presented as mean ± SEM.

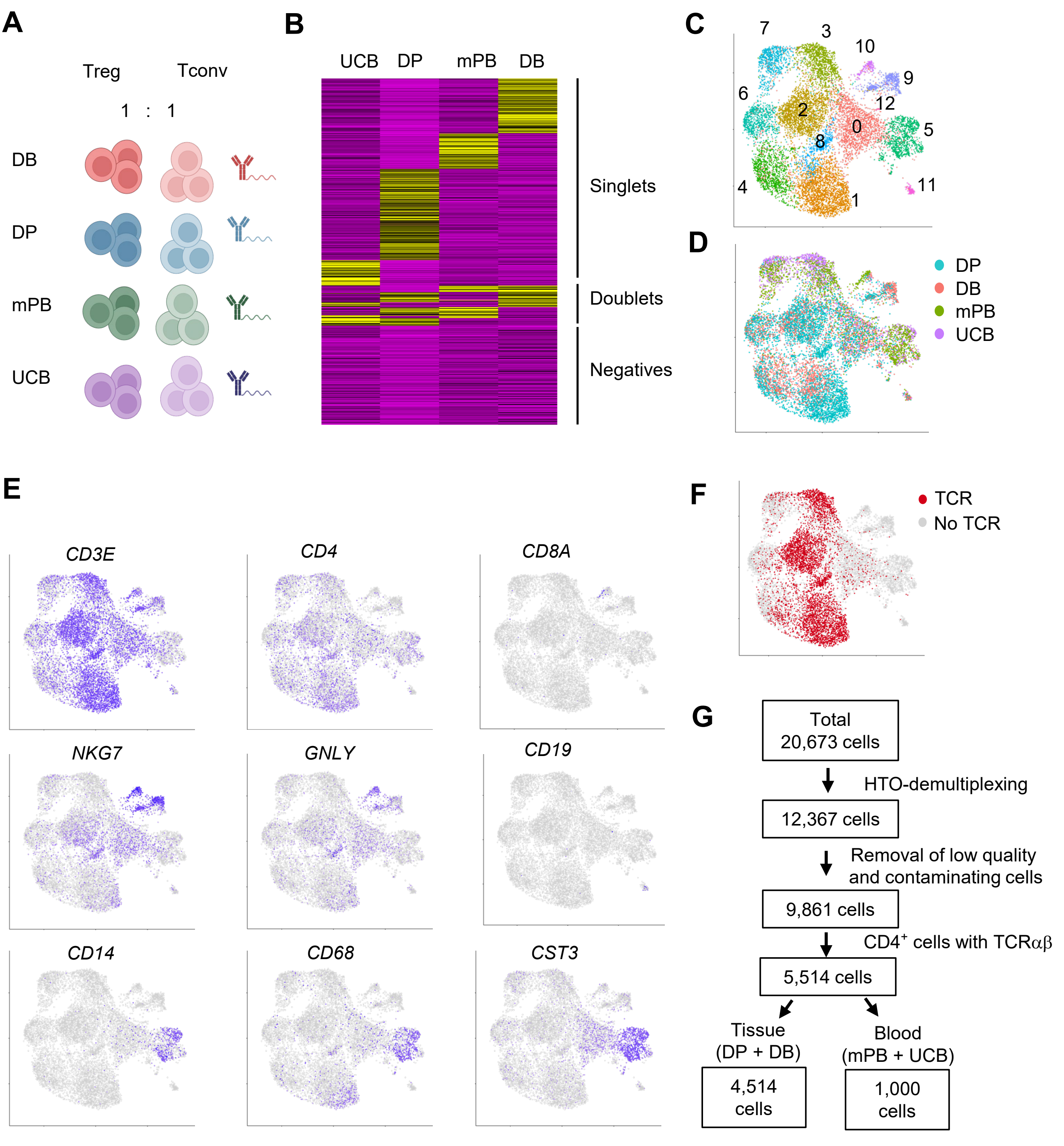

Supplementary Figure 2. Generation and (pre)processing of scRNAseq data.
(**A**) Experimental details related to the scRNAseq experiment depicted in **Figure 2**. Tregs and Tconvs were flow cytometrically sorted as outlined in **Supplementary Figure 1D** from pooled CD4^{+} T cells (n=3, see Table 1), isolated from DP, DB, mPB and UCB after term delivery by C-section. Sorted Tregs and Tconvs were pooled in a 1:1 ratio and subsequently labeled for tissue origin with distinct TotalSeq-C^{TM} antibody-coupled HTOs. Next, all samples were pooled and subjected to 10X Genomics scRNAseq coupled with scTCRseq. Created in BioRender (https://BioRender.com/ulgc5o3). (**B**) Heatmap showing HTO-demultiplexing based specific HTO signal (yellow = high; purple = low) per tissue, identifying the singlets to be included in the analysis. (**C**) UMAP projection of all single cells left in the dataset before removal of contaminating cell clusters showing cells per cluster (**C**) and per tissue-specific HTO (**D**). (**E**) Levels of marker transcripts across the UMAP of all singlets used for identification of contaminating cell clusters. (**F**) UMAP projection of singlets after removal of contaminating clusters showing cells with a retrieved TCRαβ in red. (**G**) Outline of cells remaining after each data processing step.

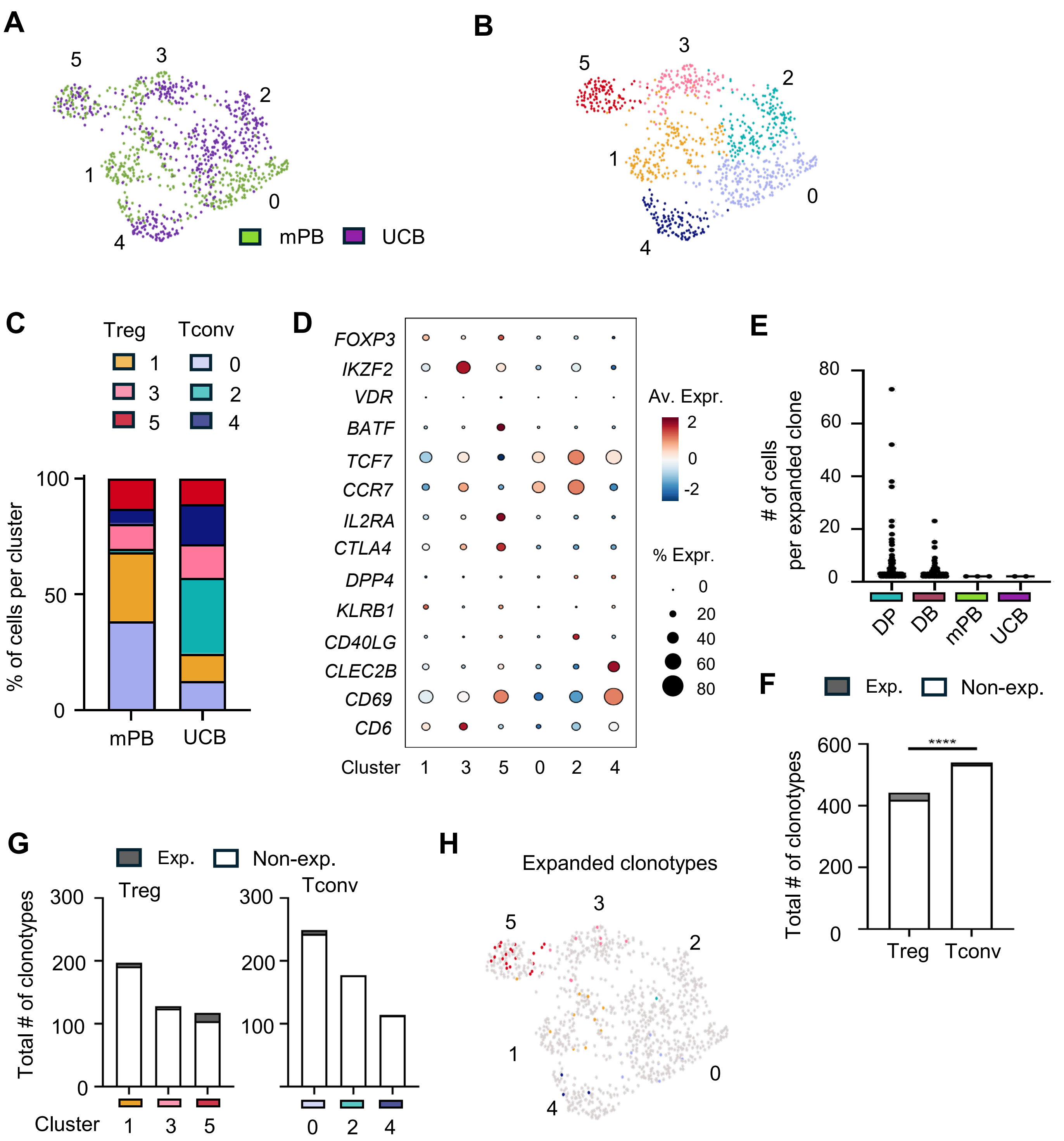

Supplementary Figure 3. scRNAseq and TCRseq of CD4⁺ T cells from mPB and UCB; identification of Treg and Tconv clusters and clonotype analysis.

CD4⁺ T cells were analyzed by scRNAseq and scTCRseq as described in Figure 2A and Supplementary Figure 2. (**A, B**) UMAP projection and clustering of CD4⁺ T cells from mPB and UCB based on the transcriptome, colored by tissue origin (**A**) and cluster (**B**). (**C**) Quantification of the cell frequency per Treg and Tconv cluster in mPB and UCB. (**D**) Quantification of levels and frequencies of transcripts of indicated Treg and Tconv marker genes per CD4⁺ T-cell cluster as defined in **B**. (**E**) Number (#) of cells in each expanded TCR clonotype per indicated tissue type. (**F, G**) Total number (#) (**F**) and number per cluster (**G**) of distinct expanded (exp.) and non-expanded (non-exp.) clonotypes identified by scTCRseq. Expanded clonotypes were defined as having ≥ 2 cells with the same TCR. (**H**) Expanded (red) and non-expanded (grey) clonotypes projected onto the UMAP depicted in (**B**). Grey dots indicate non-expanded clonotypes.

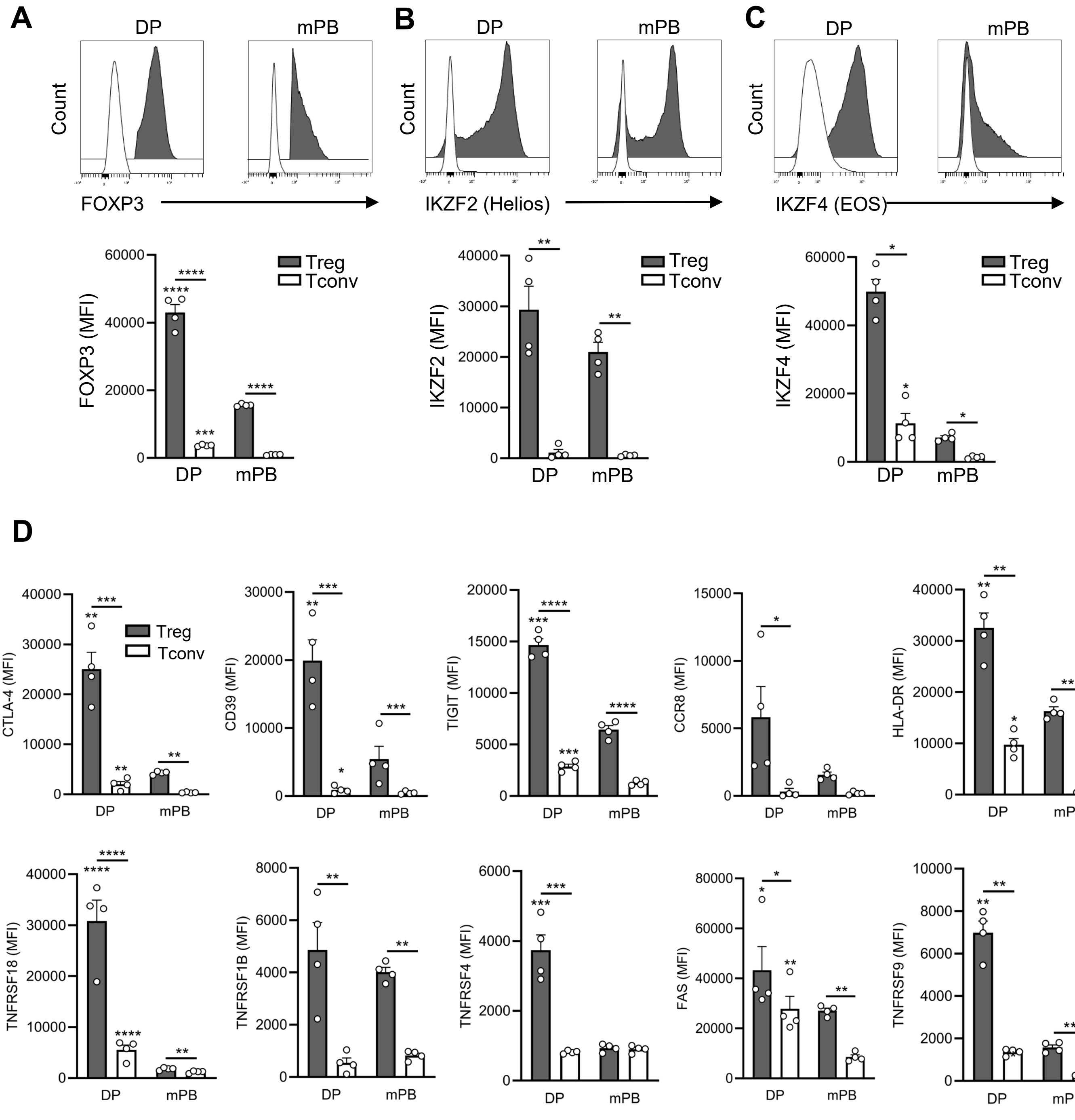

Supplementary Figure 4. Flow cytometry marker expression on Tregs and Tconvs in DP and mPB

(**A-C**) Histograms (upper panels) and quantification (lower panels) of the MFI of FOXP3 (**A**), IKZF2 (Helios, **B**) and IKZF4 (EOS, **C**) in indicated cell types, as determined by flow cytometry. Two-way repeated measures ANOVA with Tukey's post hoc test was used for statistical analysis (*p<0.05, **p<0.01, ***p<0.001, ****p<0.0001). (**D**) Cell surface protein expression and total cellular protein expression for CTLA-4, quantified as MFI of indicated receptors, as measured by spectral flow cytometry on gated Tregs and Tconvs (discriminated based on FOXP3 expression, as shown in Supplementary Figure 1). Two-way repeated measures ANOVA with Tukey's post hoc test was used for statistical analysis (*p<0.05, **p<0.01, ***p<0.001, ****p<0.0001). Data represent samples from n=4 individual donors (Table 1), presented as mean ± SEM

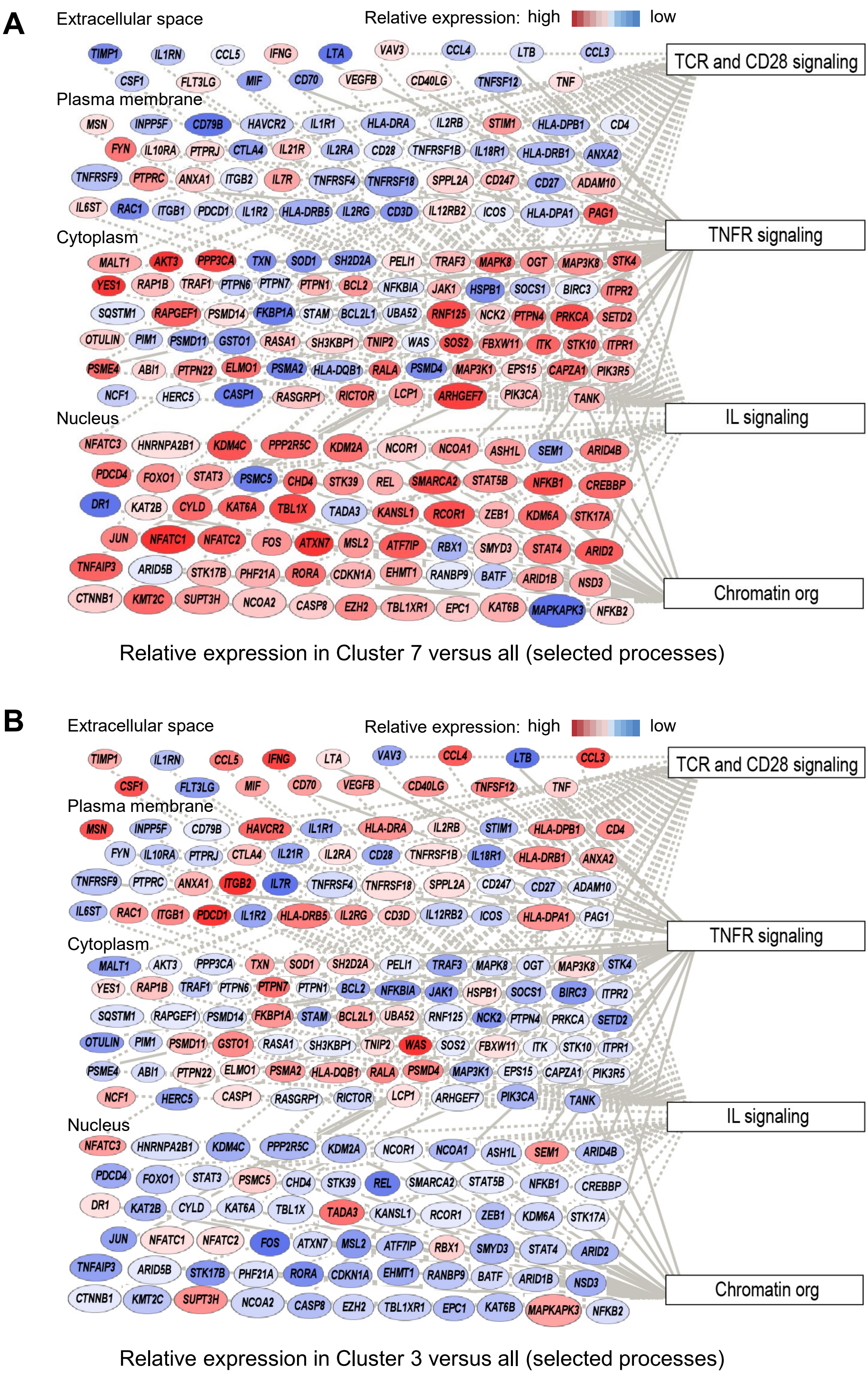

Supplementary Figure 5. Expression of signaling molecules in Tconv clusters 7 and 3.
(**A, B**) Differentially expressed genes (DEGs) associated with functional categories “TCR/CD28 signaling”, “TNFR signaling”, “interleukin signaling” and “chromatin remodeling”, are depicted according to subcellular distribution using IPA. Color scheme indicates z-scores representing transcript levels in Cluster 7 (**A**) and Cluster 3 (**B**) relative to all other clusters.

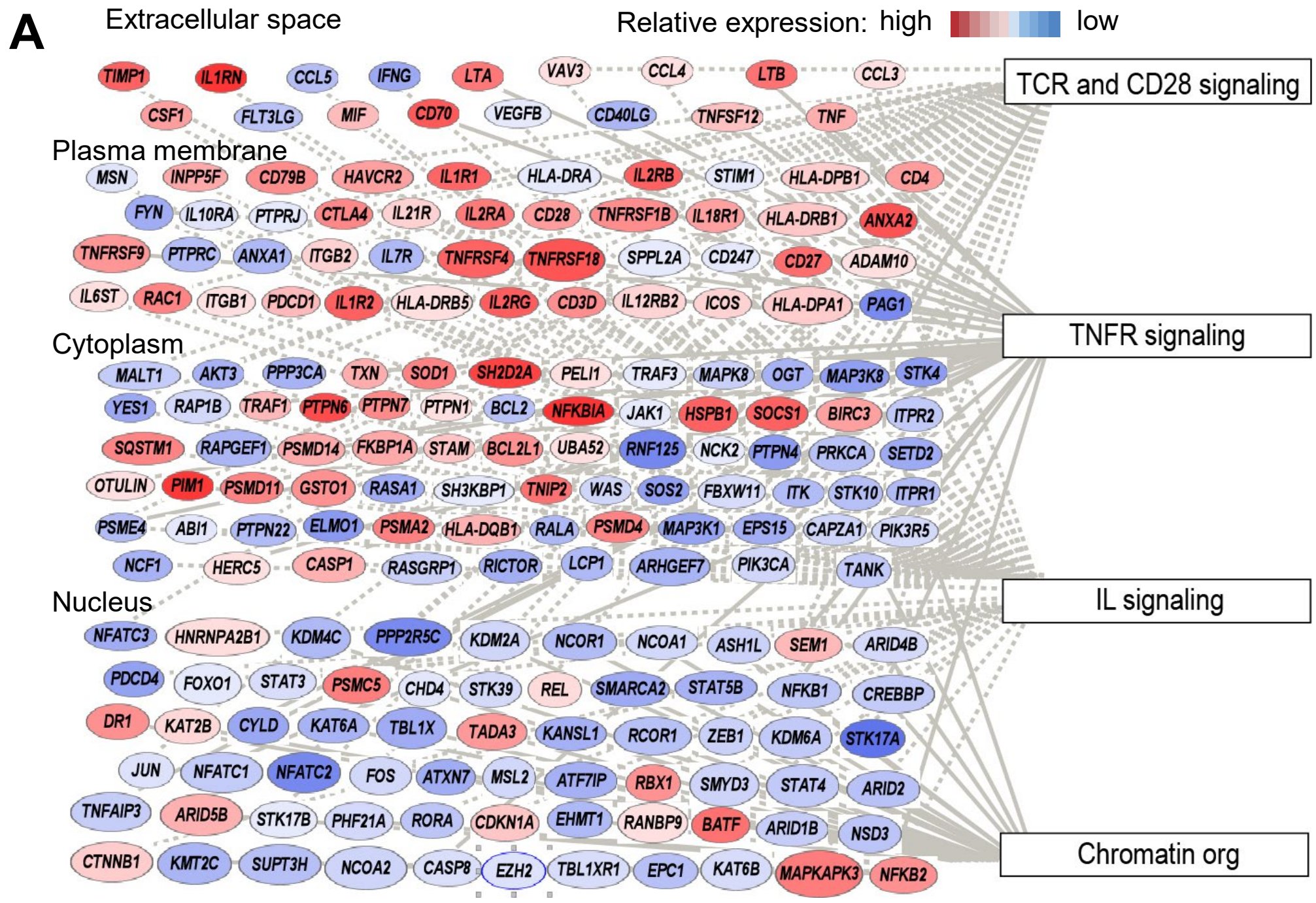

Relative expression in Cluster 2 versus all (selected processes)

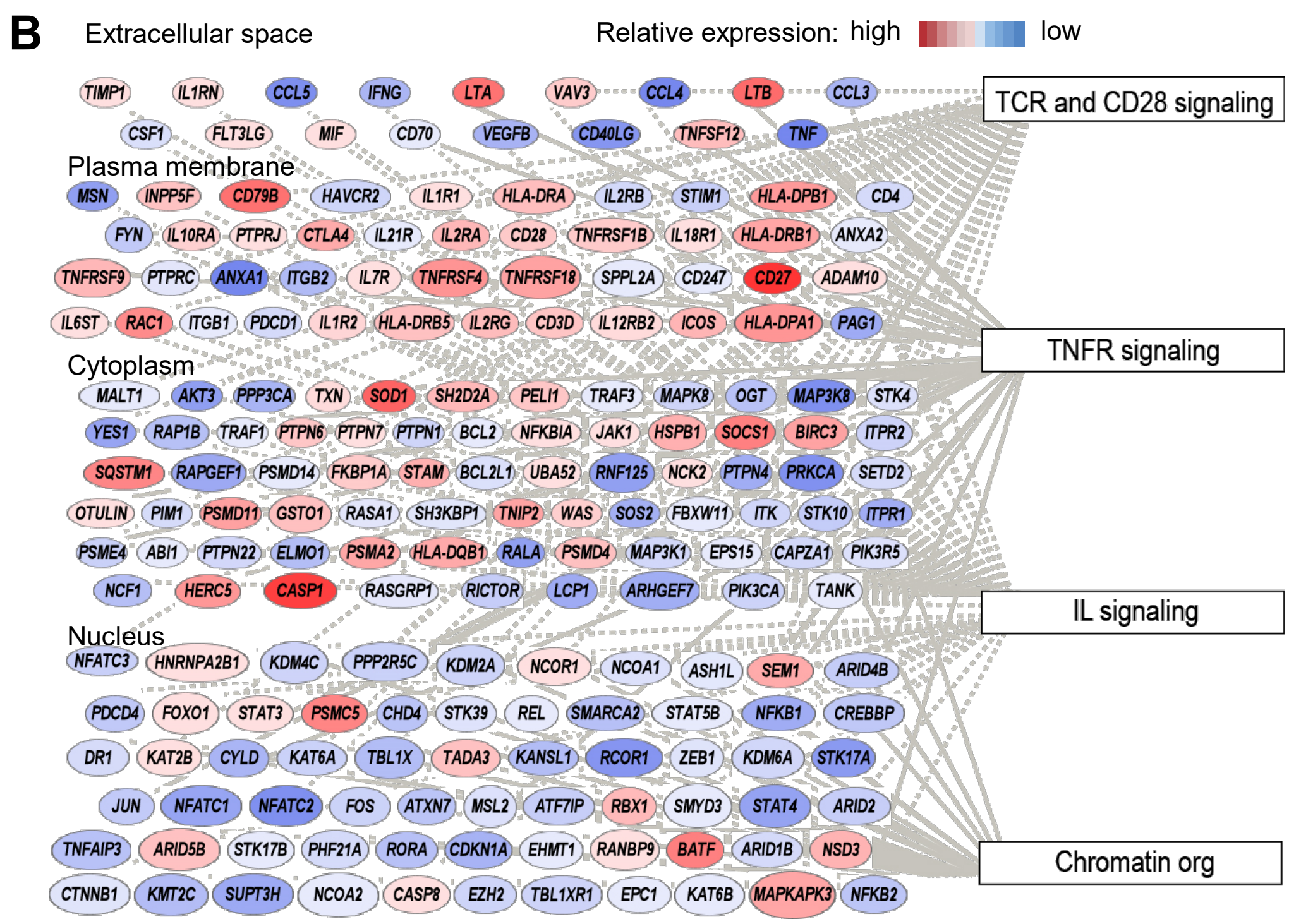

Relative expression in Cluster 1 versus all (selected processes)

Supplementary Figure 6. Expression of signaling molecules in Treg clusters 2 and 1.
(**A, B**) DEGs associated with functional categories “TCR/CD28 signaling”, “TNFR signaling”, “interleukin signaling” and “chromatin remodeling”, are depicted according to subcellular distribution using IPA. Color scheme indicates -scores representing transcript levels in Cluster 2 (**A**) and Cluster 1 (**B**) relative to all other clusters.

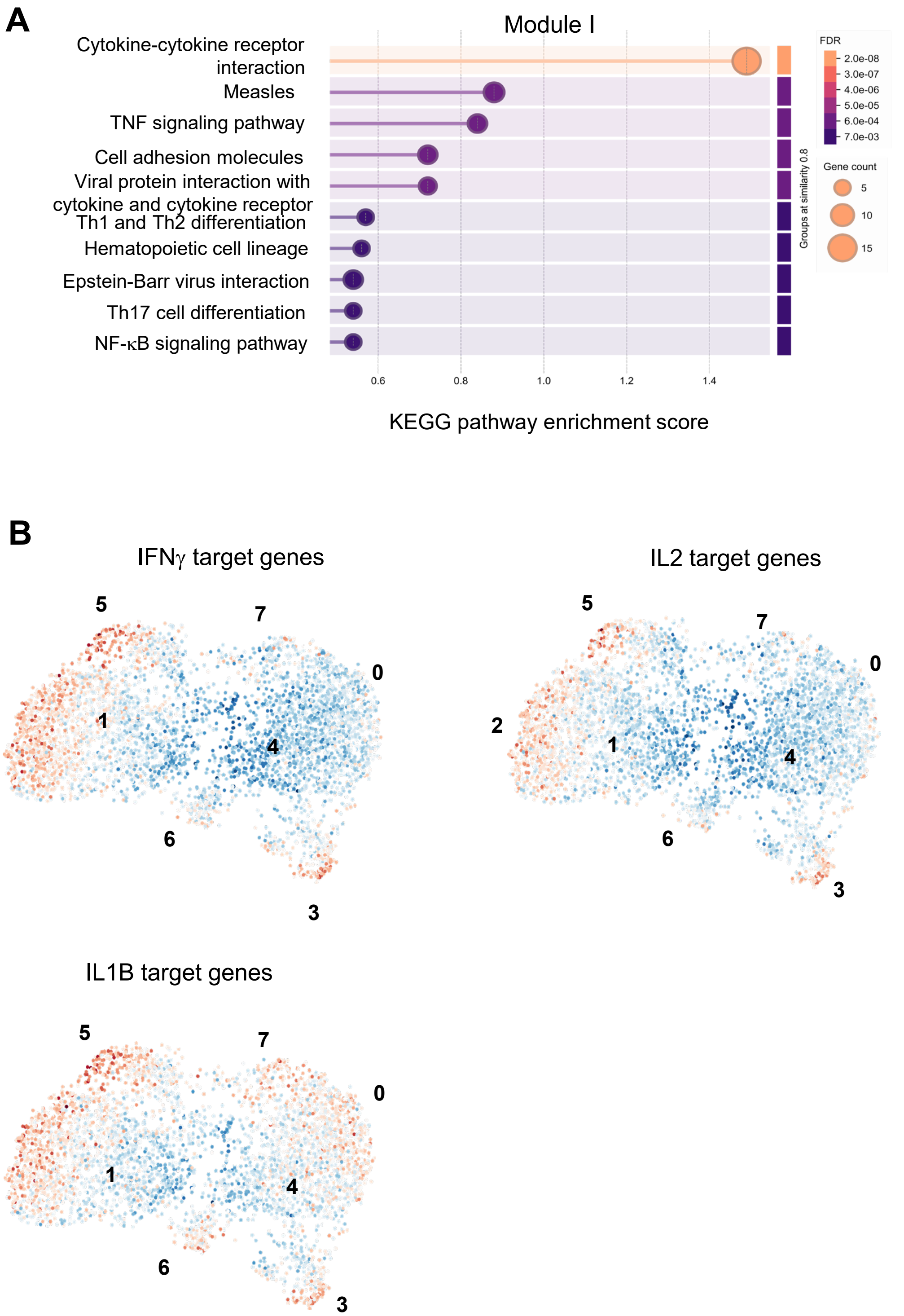

Supplementary Figure 7. Features of Module I and expression of upstream driver target genes
(**A**) Functional annotation by KEGG pathway analysis of transcripts in Module I. (**B**) Quantification of transcript levels and frequency of expression of target genes of indicated upstream drivers identified in Figure 7C, D per CD4$^+$ Treg and Tconv cluster. Color legend as for Figure 4B.
